# A Pulsatile Flow System to Engineer Aneurysm and Atherosclerosis Mimetic Extracellular Matrix

**DOI:** 10.1002/advs.202000173

**Published:** 2020-04-30

**Authors:** Vahid Hosseini, Anna Mallone, Nima Mirkhani, Jerome Noir, Mehdi Salek, Francesco Silvio Pasqualini, Simone Schuerle, Ali Khademhosseini, Simon P. Hoerstrup, Viola Vogel

**Affiliations:** ^1^ Laboratory of Applied Mechanobiology Institute of Translational Medicine Department of Health Sciences and Technology ETH Zurich Zurich 8093 Switzerland; ^2^ Institute for Regenerative Medicine (IREM) University of Zurich and Wyss Translational Center Zurich Zurich 8952 Switzerland; ^3^ Responsive Biomedical Systems Lab Institute of Translational Medicine Department of Health Sciences and Technology ETH Zurich Zurich 8093 Switzerland; ^4^ Institute of Geophysics Department of Earth Sciences ETH Zurich Zurich 8092 Switzerland; ^5^ Department of Mechanical Engineering Massachusetts Institute of Technology Boston MA 02139 USA; ^6^ Synthetic Physiology Laboratory Department of Civil Engineering and Architecture University of Pavia Pavia 27100 Italy; ^7^ Department of Bioengineering University of California‐Los Angeles Los Angeles CA 90095 USA; ^8^ Present address: Department of Bioengineering University of California‐Los Angeles Los Angeles CA 90095 USA

**Keywords:** aneurysms, arterial wall diseases, atherosclerosis, extracellular matrix, human vascular smooth muscle cells, in vitro 3D tissue models, pulsatile flow

## Abstract

Alterations of blood flow patterns strongly correlate with arterial wall diseases such as atherosclerosis and aneurysm. Here, a simple, pumpless, close‐loop, easy‐to‐replicate, and miniaturized flow device is introduced to concurrently expose 3D engineered vascular smooth muscle tissues to high‐velocity pulsatile flow versus low‐velocity disturbed flow conditions. Two flow regimes are distinguished, one that promotes elastin and impairs collagen I assembly, while the other impairs elastin and promotes collagen assembly. This latter extracellular matrix (ECM) composition shares characteristics with aneurysmal or atherosclerotic tissue phenotypes, thus recapitulating crucial hallmarks of flow‐induced tissue morphogenesis in vessel walls. It is shown that the mRNA levels of ECM of collagens and elastin are not affected by the differential flow conditions. Instead, the differential gene expression of matrix metalloproteinase (MMP) and their inhibitors (TIMPs) is flow‐dependent, and thus drives the alterations in ECM composition. In further support, treatment with doxycycline, an MMP inhibitor and a clinically used drug to treat vascular diseases, halts the effect of low‐velocity flow on the ECM remodeling. This illustrates how the platform can be exploited for drug efficacy studies by providing crucial mechanistic insights into how different therapeutic interventions may affect tissue growth and ECM assembly.

## Graphical abstract

Alterations of hemodynamics are associated with vascular wall diseases. A simple, pumpless, close‐loop, easy‐to‐replicate flow device to engineer de novo‐grown disease‐like extracellular matrix of vascular wall tissues is proposed. Mechanistic insights of a clinically practiced matrix metalloproteinase inhibitor used to treat aneurysm and atherosclerosis, and on how the stage of the disease might affect the efficacy of the treatment are gained.

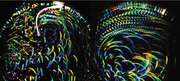

## 1 Introduction

Atherosclerosis and aneurysms represent life‐threatening pathological changes of the arterial wall,^[^ ^1^, ^2^ ^]^ and are associated with substantial morbidity and mortality caused by vessel rupture, hemorrhage, thromboembolism, ischemic events, and even leading to sudden death.^[^ ^3^ ^]^ Pathological vessel wall transformations are progressive and a regression of these conditions with noninvasive interventions is not easy to achieve. For instance, when pathological changes leading to aneurysm start to form, alterations in blood flow gradually weaken the arterial walls and cause progressive vessel expansion and finally aneurysmal wall rupture. Therefore, preventive interventional therapies are the primary choice of treatment.^[^ ^4^ ^]^ In order to improve on early diagnosis and therapy, significant challenges remain to understand the underlying mechanisms. Indeed, alterations in flow patterns (e.g., swirling flow, turbulent flow, or low velocity flow) lead to changes in stresses on thevessel wall.^[^ ^1^ ^]^ These changes induce modifications and damage of endothelial cells (ECs),^[^ ^1^, ^5^ ^]^ specifically at arterial branches, where the blood flow patterns are disturbed rather than laminar. Interestingly, it has been shown that the short exposure of confluent EC monolayers (3 h) to disturbed flow in a coin‐plate device stimulated substantial cell retraction and cell loss in vitro.^[^ ^6^, ^7^ ^]^ Even though 2D cell culture studies revealed some of the underlying mechanisms of how pulsatile flow is sensed by ECs,^[^ ^8^, ^9^ ^]^ far less is know about how the underlying smooth muscle cells (SMCs) respond to flow exposure by remodeling their extracellular matrix (ECM) once the EC layer is ruptured due to disturbed flow in vascular bifurcations and sharp bends. Collagens (mainly types I, III, IV, V, and VI) and the most abundant type I collagen are highly expressed and assembled into fibrillar networks in diseased arteries compared to normal arteries.^[^ ^10^, ^11^ ^]^ In contrast, mature elastin fibers that have a half‐life time extending to many years in humans get degraded in diseased artery walls,^[^ ^12^, ^13^, ^14^ ^]^ therefore affecting the mechanical properties of vessel walls. Although, these changes have been reported in humans and in animal models, a suitable human cell‐based 3D in vitro model to simultaneously grow diseased and healthy vessel wall and to study de novo tissue morphogenesis has not yet been introduced.^[^ ^5^, ^15^, ^16^ ^]^

**Figure 1:**
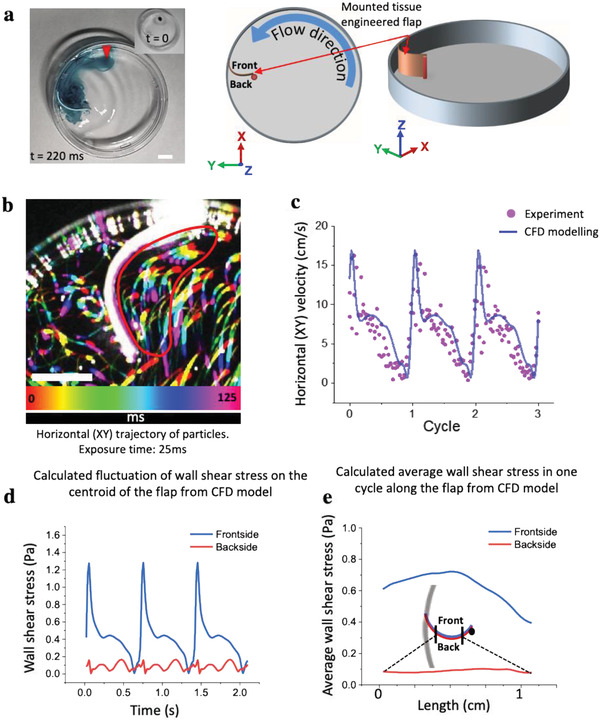
Mounting of an engineered tissue scaffold in the orbital shaker dish platform together with the CFD analysis of the oscillatory traveling wave. a) Actual image of the petri dish with a drop of ink (image inset, red arrow) to illustrate the fluid motion (Movie S4, Supporting Information), and two schemes to show how the rectangular tissue scaffold made from biodegradable PGA‐P4HB is mounted in the dish, together with defining the three coordinates used in later figures. The tissue scaffold was attached with one end to the dish wall and with the other end to an upright post, located 1 cm away from the wall, thereby creating a convexly curved barrier facing the flow direction. b) The horizontal (*XY*) trajectories of particles (compiled sequence of five images sequentially colored in rainbow colors) were visualized by short exposure (25 ms) with the aid of a high‐speed camera. The lengths of the experimental particle trajectories were used to calculate the corresponding velocities in the encircled region (as marked by the red line). Scale bars 5 mm. c) To validate the CFD calculations, the time‐resolved experimentally calculated horizontal velocities (dots) during three cycles in front of the tissue are shown to be in good agreement with the average flow velocities as obtained by CFD. d) Fluctuations of shear stresses on one spot at centroid of flap and e) average of wall shear stress along a horizontal curve on the frontside (blue line) and backside (red line) of flap in a cycle.

Although several fluidic devices were previously used to study the cell responses to flow shear stress,^[^ ^15^, ^16^, ^17^, ^18^ ^]^ to the best of our knowledge, none of them exposed de novo grown 3D SMCs tissues to physiologically relevant pulsatile shear flow conditions using a simple device. While biomimetic bioreactors driven by pulsatile pumps require complex and costly setups with limited sample throughput, we established here a simple, pumpless, close‐loop, easy‐to‐replicate orbital shaker platform capable of creating shear stresses on single bioengineered tissue. In contrast to prior orbital shaker cell culturing models^[^ ^19^, ^20^ ^]^ in which the cells were seeded on the bottom of culture dishes in plane format (2D) and were exposed to the flow at the bottom of the dish, we mounted here a tissue‐engineered 3D polymeric scaffold perpendicular to the flow direction (**Figure** **1**a; Figure S1a, Supporting Information). To set up our 3D tissue‐engineered grafts, we exploited protocols developed in the context of tissue‐engineered valvular and vascular constructs that are now in preclinical trials by using biodegradable polyglycolic‐poly(4‐hydroxybutyric acid) (PGA‐P4HB) scaffolds.^[^ ^21^, ^22^ ^]^ These 3D scaffold flaps were then mounted to the vertical dish wall and seeded with primary human SMCs with the aid of fibrinogen. The tissues were exposed to pulsatile flow as driven by the orbital shaker motion. As the concave side of the tissue faced the flow, a secondary complicated flow was created at the backside of the tissue flap.

Since the positions of disturbed blood flow region have been correlated with the development of atherosclerotic and aneurysms^[^ ^23^ ^]^ and associated with alteration of elastin and collagens,^[^ ^11^, ^24^, ^25^ ^]^ we evaluated the effects of flow on ECM formation and composition. Our device revealed that lack of pulsatile flow with association of low shear stress flow has a major influence on pathological‐like transformations of the ECM in these de novo grown tissues, as it increased the collagen and decreased the elastin content. In contrast, pulsatile high shear stress flows on the side facing to predominantly laminar flow that resembles healthy vessel wall. Since prior in vivo studies showed that ECM remodeling is mediated by matrix metalloproteinase (MMP),^[^ ^13^, ^25^, ^26^, ^27^ ^]^ MMP inhibitors were studied extensively to stop or slow down aneurysm and atherosclerosis progression with limited success.^[^ ^28^ ^]^ We, therefore, investigated the roles of various counterbalancing MMPs and TIMPs in adjusting the collagen III/I ratio and the collagen‐to‐elastin balance in this model. To illustrate the utility of this device for drug screening, we applied doxycycline, an MMP inhibitor used in clinical trials to treat aneurysmal wall pathologies, and we show that it can halt the damaging effect of the low flow velocity regime on ECM remodeling. The data suggest for the first time why doxycycline has shown limited therapeutic efficacy in the clinic; because of its role in altering the collagen I, III, and elastin balance.

## 2 Results

### 2.1 Device Setup and Quantification of the Pulsatile Flow Patterns

The rotation of the orbital shaker creates a traveling wave of fluid, which is sloshing around the center of the dish (Figure 1a; Movie S1, Supporting Information). Even though orbital shakers were used extensively to study the impact of flow on cells located at the bottom of dishes,^[^ ^20^ ^]^ the central element of our orbital shaker platform is that we mounted the tissue scaffolds perpendicular to the bottom of the dish. Therefore, one end of the scaffold was fixed to the dish wall and the other end was post‐positioned about 1 cm toward the center of the dish as sketched in Figure 1 and Figure S1a, Supporting Information. To create our device, we proceeded as follows: i) mounting of the bioengineered PGA‐P4HB microfibrillar porous scaffold (18 mm × 5 mm × 1 mm) to the dish wall; ii) seeding of a suspension of SMCs in a fibrinogen‐thrombin precursor^[^ ^29^ ^]^ into the mounted porous scaffold in order to create a provisional 3D tissue following an established prior clinical protocol;^[^ ^22^ ^]^ and iii) after a day of culture under static condition, exposing the tissue to pulsatile flow using an orbital shaker platform for 3 weeks.

**Figure 2:**
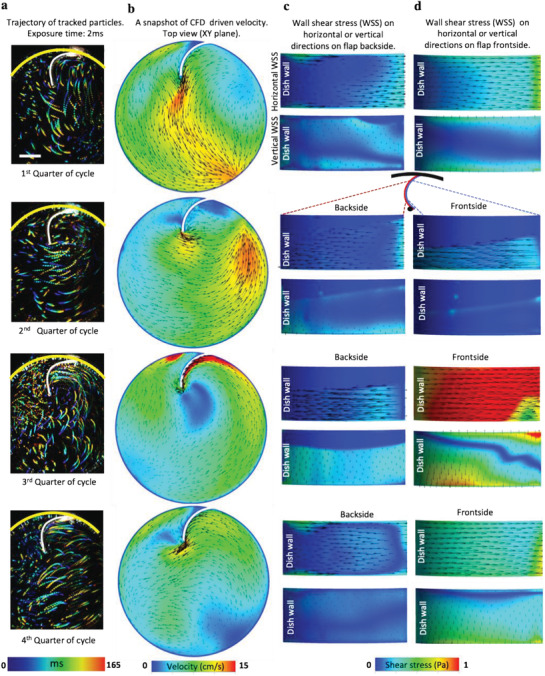
Experimental trajectories and computationally calculated velocities in horizontal (*XY*) plane beside horizontal and vertical shear stresses on the flap surface. a) Trajectories of particles visualized in four snapshots of the oscillatory cycle taken with an exposure time of 2 ms. Scale bar 5 mm. b) A snapshot of flow velocities from CFD simulations (see also Movie S4, Supporting Information). CFD analysis of shear stress tangential to the flap's curved surface in horizontal and vertical directions on the c) backside and d) frontside, respectively. The left sides of the rectangles are in contact with the dish wall and the right sides are pointing towards the center of dish (see also Movies S5–S8, Supporting Information).

To estimate the flow profiles on the *XY* plane of our open dish configuration, particularly around the tissue scaffold during each cycle, we used a combination of experimental and computational approaches to characterize the local flow profiles (Figure 1) and the flow‐induced stress (**Figure** **2**). We used laser light sheet illumination and imaged fluorescent tracer particles (38–45 µm in diameter) suspended in the medium. With the aid of a high‐speed camera, we tracked the flow near the front of the tissue experimentally using particle trajectories in the medium (Figure 1b). In a dish without the tissue, the tangential flow in the vicinity of the side wall was laminar and pulsatile (Movie S1 and Figure S1b, Supporting Information), as shown previously.^[^ ^20^ ^]^ In the presence of the tissue scaffold, complex horizontal (*XY*) flow patterns emerged, with mostly a laminar flow forming in the front of the tissue, and a secondary lower velocity disturbed flow at the back of the scaffold (Figure 1b; Movie S2, Supporting Information). To further quantify the time‐resolved flow conditions in 3D, we used the commercial ANSYS Fluent software package (ANSYS, Inc., Canonsburg, PA, USA) for computational fluid dynamics (CFD) modeling inside the orbitally shaken petri dish. We employed the commonly used *k* − *ϵ* turbulence model to simulate turbulent flow,^[^ ^30^ ^]^ where *k* represents the turbulent kinetic energy and *ϵ* is the turbulent dissipation to solve the turbulent flow. While the fluids, that is, water and air inside the petri dish, are initially at rest, it takes around six cycles of simulations until the flow reaches the periodic condition for which the results are given (Figure 1c,d,e; Movie S3, Supporting Information). In order to validate the numerical data obtained from the simulations, average horizontal (*XY*) velocity magnitudes in the front of the flap (encircled red in Figure 1b) are calculated and then compared with the experimental horizontal (*XY*) velocity data taken in the same region from particle trajectory measurements. The average velocities in the front of the flap ranged from 5 to 17 cm s^−1^ within a cycle as calculated from the encircled area, which is in the range of flow velocities found near the wall of small arteries, for example, intracranial arteries.^[^ ^31^ ^]^ In contrast, the average velocities in the back of the flap ranged from 0.5 to 5 cm s^−1^. We then used the CFD model to calculate the fluctuations of shear stresses on the centroid region of the front and backside of the flap, respectively (Figure 1d). The data indicate that the shear stresses experienced in the frontside of the flap are an order of magnitude higher than in the back, reaching a maximum of 1.3 versus 0.16 Pa, respectively. Moreover, the average shear stress during one cycle and along the flap was extracted (Figure 1e). The results show that the highest average shear stress in a cycle is felt at the middle of the flap and slightly decreases towards the two ends. On the backside, the shear stresses do not change significantly along the flap.

To visualize the flow around the dish and the obstructing tissue flap, we rendered Movie S3, Supporting Information, as obtained from CFD. The four snapshots, which correspond to four multi‐stack images that were taken by a high‐speed camera (Figure 2a,b), provide the experimental horizontal flow velocities and vectorial directions in each of the quarters of an orbital motion cycle. They illustrate that the running wave shortly hits the flap, then runs parallel to the flap in horizontal plane, thereafter departs toward the center of the dish together with the corresponding CFD snapshots of wall shear stress (Figure 2c,d). While the normal motion of flow could not be resolved from the particle trajectories, we separately analyzed the horizontal and vertical shear stress values to the dish plane from the CFD simulations (horizontal direction, Movies S5, S6 and Z direction, Movies S7, S8 in the front and back, respectively, Supporting Information). This analysis shows that the highest shear stresses are created in horizontal direction (parallel to *XY* plane) in the front, with a short vertical switch as the traveling wave hits the flap. The backside is generally exposed to significantly lower shear stress values in both horizontal and vertical directions.

### 2.2 *α*‐SMA Expression Is Enhanced at the Frontside of the Scaffold

**Figure 3:**
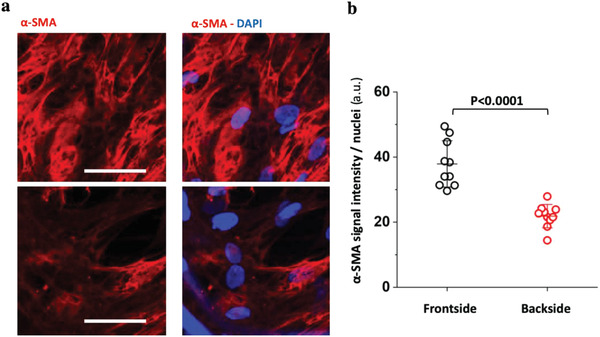
Differential *α*‐SMA content of de novo grown engineered tissues that faced either the high‐velocity or the low‐velocity pulsatile flow after 21 days of culture. a) Representative images of tissue sections that faced the high (front) versus low (back) flow velocity, respectively, immunostained for *α*‐SMA (red) and nuclei (blue). b) Corresponding pixel‐by‐pixel analyses normalized to nuclei counts revealed higher presentation of *α*‐SMA‐positive cells on side facing the high flow velocity. Minimum three images from a triplicate experiment were analyzed. Scale bar 50 µm. Statistics were calculated by one‐way ANOVA and Bonferroni post hoc test.

To increase the physiological relevance of our study, primary vascular SMCs were harvested from human vena saphenous magna vein of donors (healthy volunteer women, 40–60 years old) and were expanded.^[^ ^32^ ^]^ As describe before, vascular SMCs are transitioning to a fibroblastic phenotype in typical 2D cell culture, thus loosing SMC‐specific biomarkers such as *α*‐smooth muscle actin (*α*‐SMA) during expansion^[^ ^33^ ^]^ (Figure S2, Supporting Information). It is indeed known that vascular SMCs maintain significant phenotypic plasticity in contrast to terminally differentiated cells.^[^ ^15^ ^]^ Also in response to injury and inflammatory disease, for example, atherosclerosis, vascular SMCs undergo phenotypic modulation, characterized by decreased contractile marker expression.^[^ ^33^, ^34^ ^]^ The transition of these SMCs to a fibroblastic phenotype is, thus, partially reversible. We found that these cells significantly upregulated *α*‐SMA expression after 21 days in culture when exposed to pulsatile flow conditions (**Figure** **3**a). When facing the high‐velocity flow, immunostaining revealed 1.7 times higher levels of *α*‐SMA (Figure 3b) as well as 1.5‐fold increase in *α‐SMA* gene expression compared to the disturbed flow. This indicates a significant redifferentiation of the cells towards a SMC phenotype in the frontside compared to backside. We, thus, refer to them as vascular SMCs in this manuscript.

### 2.3 ECM Elastin Assembly Was Enhanced at the Frontside, While the Collagen I ECM Content Was Enhanced on the Backside

**Figure 4:**
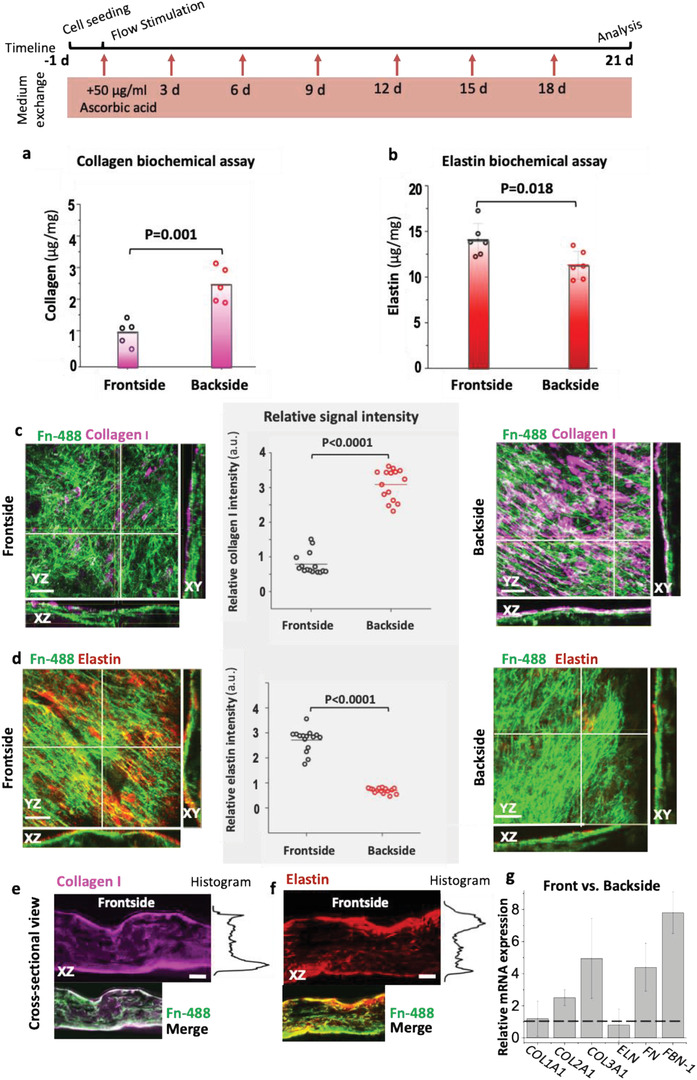
Differential deposition of collagen I and elastin in the front and backsides of the engineered tissues. The timeline summarizes how the samples have been treated from cell seeding to the analysis. a) Total acid‐pepsin soluble collagen and b) elastin concentrations were measured by biochemical assays (Sircol and Fastin, respectively) in tissues harvested from either the front or the backsides. Representative stack maximum projection intensity images of the front and backsides of tissues, which were immunostained after 3 weeks in culture for c) collagen I (magenta) and d) elastin (red). The corresponding single‐channel images are shown in Figure S2, Supporting Information. The coordinates in images are defined according to Figure 1. The outer surface layers contacting the medium used for the analyses were ≈50 µm thick. Their protein contents were assessed by comparative pixel‐by‐pixel intensity analysis of the respective immunostained images. Fibronectin was visualized not by immunostaining, but by supplementing labeled Alexa Flour 488 human plasma fibronectin (green) to the cell culture medium.^[^ ^38^ ^]^ Cross‐sectional views show the assembly of fibronectin, collagen I (e) and elastin (d) on the surface and inside the tissues. g) Relative mRNA analysis of collagens (*COL1A*, *COL2A1*, and *COL3A1*), elastin (*ELN*) at day 21. Number of biological replicates was 3 and ≥5 images were analyzed per replicate. Statistics were calculated by one‐way ANOVA, Bonferroni post‐hoc test. Scale bars c,d) 50 µm and e,f) 200 µm. See also Figures S4–S6, Supporting Information, for single‐stack confocal images of collagen I, III, and elastin, respectively.

Since diseased vessel walls are typically seen in regions of disturbed low velocity blood flow,^[^ ^23^ ^]^ such as atherosclerotic and aneurysm and associated with alteration of arterial wall ECM proteins, for example, elastin and collagens,^[^ ^10^, ^24^, ^25^, ^26^ ^]^ we evaluated the effect of flow on ECM assembly in the front and backsides of our tissue flaps. First, we assessed the total collagens (all isoforms of collagens) and elastin content on both sides of the tissues (plane *XY*, Figure 1a) by biochemical assay (**Figure** **4**a,b). To quantify the levels of collagens and elastin, we used the previously validated Sircol and Fastin reagents and protocols.^[^ ^35^, ^36^, ^37^ ^]^ By biochemical extraction, we measured significantly higher collagen levels in the tissues’ backside (Figure 4a), and vice versa, higher elastin levels in the tissues facing the high‐velocity flow (Figure 4b). Moreover, the matrix composition, including collagen I, III, and elastin, as assembled by the cells in the front and back was assessed by immunofluorescence staining. As we used identical imaging setups to analyze endpoint stains of the front and backside, pixel‐by‐pixel intensity analysis was employed to compare the relative protein assembly on each side. To visualize the fibronectin matrix, fluorescently labeled human plasma fibronectin was supplemented to the culture medium, as described previously,^[^ ^38^, ^39^, ^40^ ^]^ over the entire tissue culture with every medium exchange. The labeled fibronectin is then harvested by the cells and incorporated into their native ECM fibrils. As the tissue surfaces were uneven, labeled fibronectin was used as positional reference to also locate other ECM proteins as visualized in Figure S3, Supporting Information. Quantification of collagen I via immunostaining revealed a significant relative decrease of collagen I matrix in the front compared to the backside (Figure 4c,e; Figures S4 and S5, Supporting Information), and in agreement with the trends derived from the biochemical assay of collagen (Figure 4a). In contrast, we did not detect any relative difference for collagen III between the front and backsides of the tissues (Figures S4, S5, and S11, Supporting Information). Cross‐sectional collagen I and elastin images (Figure 4e,f) also showed that the ECM mostly formed toward the tissue surfaces, likely because of better nutrient perfusion besides the same trend of asymmetrical ECM deposition was observed. In contrast, mRNA analysis shows about equal *COL1A1* gene expression on both sides of the flap. Only the expression of *COL2A1* and *COL3A1* is significantly upregulated in the front versus back (Figure 4g).

For elastin, we see increased deposition only in the frontside (Figure 4b,d,f; Figure S6, Supporting Information), while the backside has little elastin content. This reflects well what is seen in vivo where an elastin‐rich ECM is a hallmark of healthy arteries,^[^ ^41^ ^]^ while the absence of elastin is found in degenerated ECM of disease arteries.^[^ ^13^ ^]^ In contrast to the analysis of ECM content, we did not see any differential gene expression of elastin (*ELN*) in the front versus the back. Since prior studies showed that fibronectin promotes elastin deposition^[^ ^42^ ^]^ and facilitates the assembly of fibrillin‐1^[^ ^43^ ^]^ that also promotes the assembly of elastin,^[^ ^44^ ^]^ we examine the gene expression of fibronectin (*FN*) and fibrillin‐1 (*FBN‐1*) in the front and backsides, and found 4.4‐fold and 7.8‐fold increases in gene expression of *FN* and *FBN‐1*, respectively. These findings suggest a relatively higher elastin fiber assembly in the frontside, despite the equal expression of tropoelastin, is promoted by higher expression and assembly of *FN* and *FBN‐1*. Furthermore, we performed cell nuclei density analyses on each side and found a slight increase (1.3‐fold, *p* = 0.04) in cell density on the backside (Figure S7, Supporting Information) and a significant increase of cell nuclei density in dynamically cultured tissues compared to static condition (twofold, *p* < 0.001). However, cell density alone does not explain differential ECM assembly in this model.

To see whether collagen and elastin asymmetric assembly is restricted to our specific cell type, we repeated the same experiment with primary SMCs from another human donor and with SMCs from sheep and observed similar differential flow effect on relative ECM depositions in the front and the backsides of tissues (Figures S5c,d and S6b,c, Supporting Information).

### 2.4 TIMP 1–3 Was Upregulated in the Front, But Not the Backside, Thus Inhibiting Proteolytic MMP Activity

**Figure 5:**
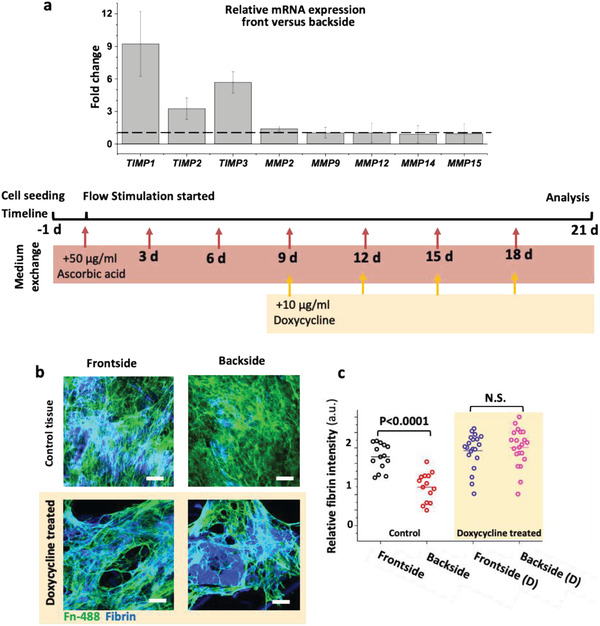
Differential mRNA levels and comparative immunostainings of the fibrin revealed that pulsatile high‐velocity flow on frontside decreased the overall MMP activity in engineered SMCs tissues by inhibiting their activity via TIMPs after 21 days in culture and doxycycline inhibit the fibrin remodeling by inhibition of MMPs. a) Relative mRNA expression of metalloproteinase inhibitors (*TIMP1*, *2*, and *3*) and MMP (*MMP 2*, *9*, *12*, *14*, and *15*) at the frontside versus backside. b) Comparative confocal microscopy and c) pixel‐by‐pixel intensity analysis of images showed delayed degradation of fibrin in the tissue's front versus backside in the absence or presence of doxycycline (10 µg mL^−1^). Fibronectin was visualized not by immunostaining, but by supplementing labeled Alexa Flour 488 human plasma fibronectin (green) to the cell culture medium.^[^ ^38^ ^]^ Number of biological replicates was 3, ≥5 images were analyzed per replicate. Flow diagram shows how the samples have been treated during the culturing period. Scale bars 50 µm. See also Figures S8 and S9, Supporting Information, for respective single channel and cross‐sectional confocal images.

To reconcile the differences between the observed ECM deposition as quantified by immunostaining and/or biochemical assays and the transcription mRNA levels of ECM proteins, we asked whether tissue metalloproteinase (MMPs) and their inhibitors (TIMPs) play a role in the ECM remodeling as observed here. Prior studies have described the role of MMPs and TIMPs in aneurysm progression, rupture,^[^ ^28^, ^45^ ^]^ and atherosclerosis^[^ ^46^ ^]^ showing the importance of these regulators in vascular pathology. TIMPs members also have been shown to inhibit several disintegrin metalloproteinase (ADAMs). Particularly TIMP‐3 acts as inhibitor of the procollagen N‐proteinase (ADAMTS‐2) and is therefore a key regulator of collagen fibrillogenesis.^[^ ^47^, ^48^ ^]^ To see how the MMPs‐TIMPs balance might affect our 3D model tissues, we conducted RT‐PCR analyses of mRNA isolated from each side (exposed to different flow conditions) after 3 weeks of culture (*N* = 3). We found that the mRNA expression of *MMPs* in general was not significantly affected in the front versus the backside (**Figure** **5**a). In contrast, *TIMP 1* and *3* showed a significant upregulation in the front versus backside (Figure 5a).

To further assess the total protease activity of cells on each side of the bioengineered 3D vascular wall, we checked fibrin degradation as an indirect indicator for protease activity^[^ ^46^ ^]^ by immunostaining and comparative signal intensity analysis (Figure 5b,c; Figure S8, Supporting Information). In comparison to the front, we observed higher fibrin degradation, indicating a higher protease activity rate at the backside. These findings suggest that the pulsatile laminar flow in the front shifts the MMPs/TIMPs balance in favor of protecting the ECM of the de novo grown SMCs tissues, by inhibiting the proteolytic activity of the SMCs.

### 2.5 Doxycycline, a Controversial Clinical MMPs Inhibitor, Prevents ECM Degradation and Remodeling under Disturbed Pulsatile Flow

In a proof‐of‐concept study, we asked whether our platform is suitable to gain insight into the efficacy of drugs on 3D tissues grown from human cells. Thus, we investigate the effect of doxycycline, a general MMP inhibitor,^[^ ^49^ ^]^ yet controversial drug that has been used in clinical trials to treat aneurysm and other vascular diseases.^[^ ^50^, ^51^, ^52^ ^]^ While doxycycline‐treated tissues are exposed to flow in in vivo models, all in vitro tests were performed in static cell culture models,^[^ ^53^, ^54^ ^]^ and the drug was never tested in a dynamic in vitro model to the best of our knowledge. To further clarify the role of MMPs, we thus assessed the efficacy of doxycycline on ECM remodeling by SMCs and asked how MMP inhibition by doxycycline alters the asymmetric deposition of collagen, elastin and degradation of fibrin under pulsatile flow conditions. We assessed the fibrin content of the tissues, in the presence and absence of doxycycline at the concentration of 10 µg mL^−1^, in the same range of blood serum concentrations of patients that were clinically treated to manage aneurysm growth.^[^ ^55^ ^]^ We found that the accelerated fibrin degradation was halted upon doxycycline treatment on the tissue backside, reaching the same levels as for the tissues facing the high velocity flow (Figure 5b,c; FigureS9, Supporting Information).

**Figure 6:**
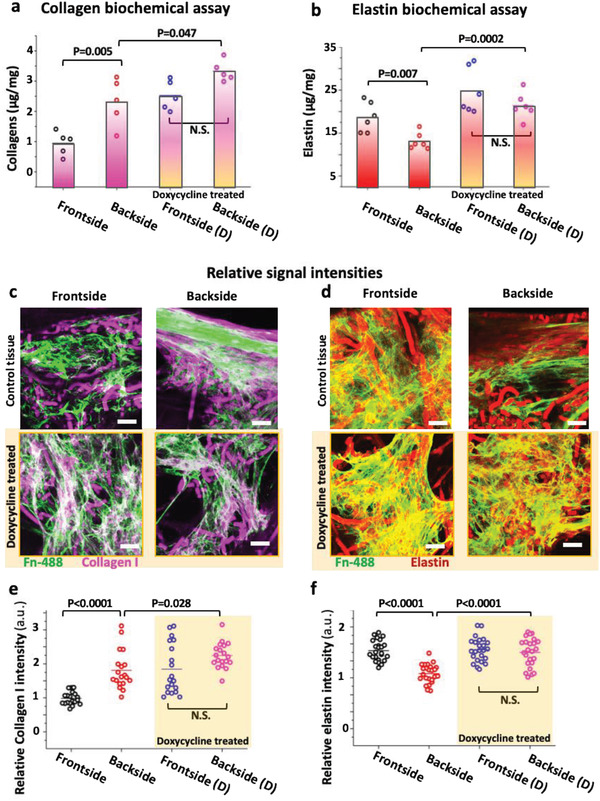
The MMP inhibitor doxycycline enhances collagen I and elastin accumulation in both of the engineered SMCs tissues. Biochemical assay of total a) collagen and b) elastin revealed higher assembly of collagen within the backside tissues and higher elastin content within the frontside tissues, but the effect of low‐velocity flow reduced in the presence of doxycycline (10 µg mL^−1^, yellow‐shaded bars) after 3 weeks of culture. Comparative immunostaining of c) collagen I (magenta) and d) elastin (red) in the presence and absence of doxycycline. Fibronectin was visualized not by immunostaining, but by supplementing labeled Alexa Flour 488 human plasma fibronectin (green) to the cell culture medium.^[^ ^38^ ^]^ Relative signal intensity analysis of e) collagen I and f) elastin showed the same trend as found in biochemical assays. Number of biological replicates was 3; ≥5 images were analyzed. All tissues have been treated according to the timeline given with the Figure 4e. Scale bars 50 µm. See also Figures S10–S12, Supporting Information, for single‐channel confocal images of collagen I, III, and elastin, respectively.

To quantify how ECM formation is affected by the presence of doxycycline (10 µg mL^−1^), we quantified collagens and elastin (*N* = 6 each). In the doxycycline‐treated tissue's frontside compared to the non‐treated controls, the total collagen content is ≈2.5 higher (**Figure** **6**a). In the collagen‐rich backsides, doxycycline treatment further increased the collagen content by ≈1.4‐fold (Figure 6a). The same trend is seen using the pixel‐by‐pixel analysis of the ECM images (Figure 6c,e; Figure S10, Supporting Information), yet less pronounced.

Remarkably, even though collagen III did not show flow‐dependent difference in the non‐treated group, its deposition in the tissues facing the front was significantly increased in the presence of doxycycline (*p* < 0.0001, Figure S11, Supporting Information). Finally, doxycycline significantly improved the elastin content on both sides and reduced the asymmetrical difference to nonsignificant value (Figure 6b,d,f; Figure S12, Supporting Information). Our findings thus show that doxycycline treatment did suppress the asymmetry, and slightly improved the total elastin deposition in contrast to the controls, thus confirming the counterregulatory roles of MMPs in disrupting elastin assembly. Taken together, our data suggest that TIMPs are the major drivers that steer the differential ECM compositions in our de novo grown tissues. These findings further highlight the necessity to include pulsatile flow conditions in the in vitro studies. It is also remarkable that the de novo grown tissue cultured for 3 weeks under flow conditions effectively recapitulate ECM compositions found in healthy and pathological human blood vessels. Besides, these data once more point to the major role of flow on SMCs and their capacity to tweak the TIMPs/MMPs balance to assemble and remodel ECM.

We also tested doxycycline at a concentration of 40 µg mL^−1^, the same concentration that has been used in prior animal and in vitro cell studies.^[^ ^53^, ^54^, ^56^, ^57^ ^]^ At such a high concentration, doxycycline completely prevented SMCs to remodel and digest the fibrin hydrogel in the tissue (Figure S13, Supporting Information). Additionally, a comparatively small amount of ECM was produced (Figure S13, Supporting Information) and the cells were not able to expand and migrate out from the fibrin matrix, although after removing the drug cells were recovered and expanded (Figure S14, Supporting Information). Our data thus suggest that doxycycline in such animal and in vitro studies might have been overdosed and completely suppressed the SMCs activity.

## 3 Discussion

While a number of pump‐driven in vitro fluidic cell culture models have been proposed for vascular research,^[^ ^15^, ^17^, ^58^ ^]^ we introduce here a platform that can impose simultaneously two distinct pulsatile flow conditions on bioengineered 3D tissues (Figure 1). Our device is different from other often utilized cell culture models^[^ ^19^, ^20^ ^]^ that use the bottom of dish to impose a flow stress on 2D culture of cells. Here, our tissue‐engineered flap was mounted perpendicular to the bottom, thus facing and redirecting the flow, thereby exposing the tissues to horizontal and normal force components, which are poorly mimicked in common model devices (Figure 1a). While this design allows to simultaneously investigating the impact of two flow regimes, creating comparable flow conditions within tubular 3D bioengineered tissues is much more difficult to accomplish. For instance, a prior study showed that laminar pulsatile flow induced the assembly of elastin on the luminal side of the tubular engineered vessel,^[^ ^37^ ^]^ but no data were provided on how more turbulent flow patterns might impact the outcome. Because of this novel design, our device enabled us to show the differential effects of flow on the opposing sides of in vitro grown 3D tissue and we quantified flow velocities and shear wall stresses (Figure 2). Since the EC layer is disrupted in pathological vascular tissues thereby exposing the SMCs to disturbed flow,^[^ ^59^ ^]^ we studied here the direct effect of flow on the de novo tissue growth of SMCs. Human SMCs were seeded into PGA‐P4HB‐fibrin 3D scaffolds and cultured for 3 weeks under pulsatile flow conditions. As native SMCs are not equipped with shear sensors, like ECs, much less is known how flow exposure might alter the physiology of SMCs. For the first time in an in vitro 3D engineered tissue model, we show here that altered fluid flow conditions can have major impact on the ability of SMCs to assemble and remodel ECM (Figure 4). These overall changes in ECM composition are accompanied by significantly upregulated levels of *α*‐SMA at the gene and the protein expression (Figure 3), indicating increased redifferentiation toward the SMC phenotype when exposed to high‐velocity flow as discussed before. In the high‐velocity front versus low‐velocity backside of the flap, the ECM is significantly enriched in elastin, while the collagen I content is decreased (Figure 4a,c,d,e). In contrast and at first surprising, *COL1A1* and *ELN* gene expression is mostly equal in the front and backsides of the flap (Figure 4g). Even more drastic, the *COL3A1* gene expression is increased fourfold in the front, yet the total collagen content of ECM in the back is significantly higher in the back (Figure 4a). Taken together, this suggests that the proteolytic activities as tuned by the flow‐exposed SMCs in the front versus back might be significantly different as verified here (Figures 5 and 6).

**Figure 7:**
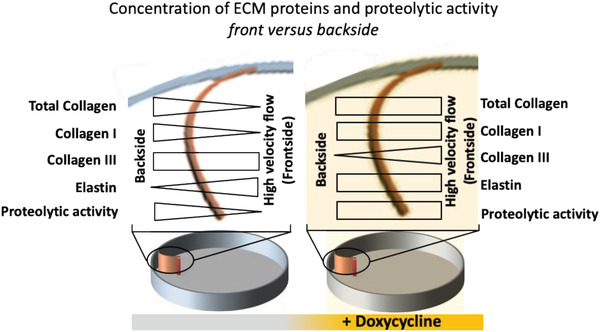
Summary of relative ECM compositions as deposited in de novo grown tissues in response to different flow regimes, as well as in the presence of the MMP inhibitor doxycycline, a drug that is clinically used to treat aneurysm and atherosclerosis plaques. In the upper part, the top views of the curved engineered tissues attached to the edge of the device platform are shown.

The differential changes of ECM compositions as summarized in **Figure** **7**, namely increase assembly of collagen and loss of elastin in these de novo grown tissues (Figure 4), thus recapitulate ECM transformations reported from vascular aneurysm and atherosclerotic tissues.^[^ ^10^, ^60^ ^]^ Our de novo grown tissues, thus, assembled an ECM under pulsatile high‐velocity flow that displayed some hallmarks of those characteristic for healthy arteries, even though SMCs under healthy physiological conditions are not directly exposed to flow. This suggests that the elastin production per se might not be affected by exposure of SMCs to high velocity flow, but that elastin degradation is only induced when SMCs get exposed to disrupted low velocity flow.

Shifting the TIMPs/MMPs balance is known to play a major role in vascular diseases such as aneurysm, atherosclerosis, hypertension, calcification of heart valves, and of other valvular remodeling diseases.^[^ ^45^, ^61^, ^62^ ^]^ Even though different clinical and animal studies showed a higher MMP expression and activity in pathologic arterial wall tissues,^[^ ^45^, ^63^, ^64^ ^]^ we did not find significant changes in *MMPs* gene expression of SMCs tissues in the front and backsides (Figure 5a). Instead, the expression of *TIMPs 1* and *3* was significantly upregulated in the tissues facing the pulsatile traveling wave, which we now propose is protecting ECM degradation. As this suggests enhanced proteolytic activity within the backside of the tissues, we further tested this hypothesis by supplementing the medium with the doxycycline, which is a general MMP inhibitor.^[^ ^49^ ^]^ Indeed, doxycycline halted the proteolytic activity of SMCs as shown by a fibrin degradation assay (Figure 5b), thus confirming our conclusion that a relatively higher elastase activity coupled with a lower TIMPs activity disrupts elastin assembly in the backside (Figure 4). We further propose that the relatively higher gene expression of *FN* and *FNB‐1* (Figure 4g) could further enhance the elastin assembly in the front.^[^ ^42^, ^43^ ^]^

Most importantly with respect to clinical applications of metalloproteinase inhibitors, treatment with doxycycline resulted in an overall increase in collagen and elastin deposition and reduced the differential effect of our two flow conditions on the total collagen and elastin ECM content (Figure 6). In this context, it is interesting to note the 5.6‐fold upregulated *TIMP‐3* expression in the front versus the backside (Figure 5a), as TIMP‐3 is the only member of TIMPs that inhibits the procollagen N‐proteinase (ADAMTS‐2). ADAMTS‐2 is responsible for cleaving procollagen C‐propeptides as required for collagen fibrillogenesis.^[^ ^47^, ^48^ ^]^ This suggests that the higher expression of *TIMP1‐3* protects the assembled ECM fibers, while at the same time, inhibits the assembly of collagens in the frontside of the flap. Remarkably, doxycycline treatment reduced the relative expression of *TIMP‐3* from 5.6‐fold to 1.5‐fold (front compared to backside), thereby confirming the role of TIMP‐3 on collagen assembly in our drug‐treated tissues.

Two possible causes of aneurysm rupture are well described in the literature:^[^ ^3^, ^10^, ^25^, ^26^, ^45^, ^59^ ^]^ i) increased proteolytic activity of the cells in vessel walls; and ii) progressive remodeling and imbalance of the ECM content. Since doxycycline was subjected to several human clinical and animal preclinical studies to treat a variety of vascular diseases^[^ ^50^, ^51^, ^52^ ^]^ and showed controversial effects in the management and prevention of aneurysm,^[^ ^28^, ^45^ ^]^ we finally asked what can be learned from our model regarding the efficacy of doxycycline. Doxycycline switched the symmetric ECM fibril deposition of collagen III to asymmetric deposition, with decreased levels at the back of the tissues (Figure S11, Supporting Information). Previous in vitro experiments suggested that alterations of the tropocollagen types I and III ratio change the mechanical properties of the heterotypic collagen fibrils with different mixing ratios of tropocollagen III to I, whereby coassembly of collagens I and III caused the fibrils to be thinner with a shorter D‐banding than pure collagen I, resulting in a higher elastic modulus.^[^ ^65^ ^]^ Taken together with our data, this suggests that the accumulation of collagen I^[^ ^11^ ^]^ ultimately decreases the collagen III/I ratios (Figure 7), implying that this might contribute to a stiffening of the vessel wall. Collagen III/I ratios were reported to be higher in soft and elastic tissues compared to stiffer tissues^[^ ^65^ ^]^ and patients with collagen III deficiency have an increased risk of cerebral aneurysm rupture.^[^ ^66^ ^]^ Our findings shed new light into the controversial discussion of the efficacy of doxycycline, which we now propose depends on the progressive stage of vascular diseases. In a highly flow‐dependent response, doxycycline treatment can have an either positive or negative effects on vascular ECM remodeling (Figure 7). On the beneficiary side, doxycycline might halt the ECM degradation by reducing the proteolytic activity of SMCs, as seen clinically,^[^ ^61^ ^]^ and thus protect the existing ECM fibrils. On the negative side, treatment with doxycycline could increase the collagen I content and thereby reduce the collagen III/I ratio in the presence of disturbed low‐velocity flow, which subsequently might lead to a stiffening and loss of elasticity of the vessel wall and thereby increase the risk of rupture.

In conclusion, treating our tissue engineered flaps with doxycycline might provide critical new insights why the clinical data are ambivalent.^[^ ^28^, ^45^ ^]^ Our data suggest that the critical balance of beneficiary and harmful effects might depend on the progressive stage of vessel wall pathology. Aneurysm therapy with general MMP inhibitors in late stages of the disease, when the majority of elastic fibrils have been already degraded, might not be effective: doxycycline treatment might then even stiffen the vessel wall by changing the collagen III/I ratio, thus enhancing the risk of wall rupture. However, in early stages, where the majority of elastic fibrils are intact, doxycycline might prevent degradation of existing ECM and slow down the disease progression. Thus, prior to administration of doxycycline or other MMP inhibitors, we propose that the stage of the disease must be screened carefully to prevent such an adverse effect and to improve the therapeutic outcome. Beyond these significant insights into how SMCs respond to different flow conditions, our data also show that our device is well suited to test the efficacy of drugs on tissues grown from human cells, and that mechanisms leading to side effects can, thus, be studied under in vitro conditions. When combined with autologous cells, this illustrates how the platform can be exploited in the future for drug efficacy studies for personalized medicine applications, giving mechanistic insights into how different therapeutic interventions might affect tissue growth and ECM assembly. It thus has significant potential for applications in personalized and precision medicine.

## 4 Experimental Section

##### Scaffold Preparation and Device Assembly

Commercially available nonwoven PGA scaffolds (thickness 1.0 mm; specific gravity 70 mg cm^−3^; Cellon, Luxembourg) were coated with 1% P4HB (MW: 100 000; Tepha, Inc., MA, USA) in tetrahydrofuran (Sigma‐Aldrich, Switzerland).^[^ ^67^ ^]^ Thereafter, the scaffold was cut into rectangular pieces (5 mm × 18 mm) that were fixed on one end to the inner wall of a petri dish, and on the other end to a post 1 cm inside the dish using an UV optical glue (Norland Products, Inc., NJ, USA) according to the detailed plan given in Figure S1, Supporting Information, to reproduce the curvature. The final constructs were sterilized using a mixture of 10% hydrogen peroxide in 70% ethanol and UV light for 1 h. The installed scaffolds were then washed at least three times with sterile phosphate‐buffered saline (PBS), followed by overnight soaking in culture medium consisting of Dulbecco's modified Eagle's medium GlutaMax (Invitrogen, USA) supplemented with 10% fetal bovine serum (Invitrogen) before cell seeding. In the second scenario and to assess the overall ECM content of tissues facing either the laminar or the disturbed flow quantitatively, two similar sheets of PGA‐P4HB scaffold were placed together and installed into the culture dish using the same configuration and sterilization methods as mentioned above. To carefully analyze the tissue formation processes at the front and backsides, the flaps consisted of two scaffold sheets sandwiched on each other, such that they could be peeled apart after cell culturing and processed separately for proteins and gene expression analyses.

##### Flow Velocity Measurements in Orbital Shaker Platform Using Particle Image Velocimetry

The velocity of the flow circulating around the petri dish in a horizontal plane 3 mm above the bottom of the petri dish was measured with illuminating laser light sheet (550 nm) according to the previously described method.^[^ ^20^ ^]^ To do this, fluorescent polyethylene microspheres (38–45 µm, Cospheric, USA) were dispersed in water and placed into the petri dish at height of 10 mm. The entire setup, including laser light source and camera, was assembled on the orbital shaker platform to capture the flow and not the overall movement of petri dish. A high‐speed camera was used to capture images at different exposure times. To estimate the velocity in the region of interest (near the wall, back and front of scaffolds), strike length of particle track was measured in images. The velocity of particle was calculated by using an exposure time of 25 ms with an acquisition rate of 37 images per second. The average velocity at each time point was calculated by averaging measured particles velocities in the region of interested. Also, to visualize the particle movements in the whole field of view (Figure 1d), high‐speed images with acquisition rate of 110 image per second and 2 ms exposure time were captured, then superimposed and temporary color‐coded using ImageJ software and presented in quarter of one cycle.

##### Computational Modeling

ANSYS Fluent (ANSYS, Inc.) was used to computationally model the 3D unsteady two‐phase flow inside the orbitally shaken petri dish, that is, a traveling water wave in contact with the gas phase as confined by the circular geometry of the petri dish. The flap geometry was modeled as a fixed solid boundary with average thickness of 800 *μ*m mounted to the inside wall of the petri dish which was initially filled with 8 mm height of water as the liquid phase while air occupied the rest of the volume. The commonly used *k* − *ε* turbulent model was employed to simulate circular turbulent flow,^[^ ^30^ ^]^ where *k* represents turbulent kinetic energy and *ε* represents the turbulent dissipation to solve the turbulent flow governing equations, that is, Reynolds‐averaged Navier–Stokes equations. By using a frame as a reference, motion of the orbital shaker was prescribed for the whole domain representing motion of the dish with respect to a static observer. Movement of the shaker was exerted to the whole domain with dynamic mesh interface in the software package. User‐defined function written in C++ language was compiled to implement the rigid body motion of the shaker. The free‐surface between the two phases was captured using volume of fluid method in which single set of momentum equations is solved for both phases and volume fractions are tracked through the domain. To represent the time‐dependent shape of the interface, a geometric reconstruction scheme was adopted according to the ANSYS Fluent Theory Guide (release 15.0.2013; ANSYS, Inc.). Water was modeled with a density of $\rho =998\frac{\mathrm{kg}}{m^{3}}$ and viscosity of *η*  =  1e − 3 Pa s and a surface tension of 45 mN m^−1^. Air properties were assumed to be $\rho =1.2\frac{\mathrm{kg}}{m^{3}}$ and *η*  =  1.8e − 5 Pa s. No slip condition was applied to all wall boundaries.

##### Cell Isolation, Seeding, and Culturing Condition of Scaffolds

Human venous vascular SMCs were harvested from human vena saphenous magna donors (healthy volunteer women, 40–60 years old) according to the Zurich University Hospital ethical approval (KEK‐Stv‐21‐2006) and expanded as described before.^[^ ^32^ ^]^ Cells with passage number between 4 and 8 were used in this study. Cell seeding was performed using fibrin gel as carrier as previously described.^[^ ^68^ ^]^ Briefly, cells were defrosted, expanded, and trypsinized based on standard cell culture protocols. Then cells were resuspended in a sterile bovine thrombin (Sigma, USA) solution in PBS (10 IU thrombin per mL). Fibrinogen solution was prepared by dissolving 14 mg of lyophilized bovine fibrinogen powder (Sigma) equal to 10 mg mL^−1^ active fibrinogen into the culture medium then was sterilized using 0.21 µm sterile syringe filter. Subsequently, the cells in the thrombin solution were added to a sterile bovine fibrinogen in equal volume. After carefully mixing, the fibrin solution containing the cells was dripped onto the scaffold to fill the scaffold porosities. For double‐layer constructs, double number/volume of cell‐fibrinogen solution added to scaffolds and let them set. The coagulation time of the fibrin gel was determined to vary from 20 to 40 s. After the seeding procedure, constructs were kept for polymerizing for 15 min in the incubator before addition of the medium. All the constructs were placed in static conditions for 24 h; at this point, the mechanical stimulation was started using an orbital shaker (VWR International, USA). The rotation radius of the shaker was 19 mm and the rotation speed was set to 1.5 Hz (90 rpm). Culture medium was exchanged every 3 days, and the incubation was performed at 37 °C, 10% CO_2_. Cell passages from 4 to 8 were used in this study.

##### Total Collagen Analysis

The Sircol soluble collagen assay kit (biochemical, colorimetric method from Biocolor, UK) was used to analyze the total collagen content of tissues.^[^ ^69^ ^]^ To do this, the tissue samples were cut and washed with PBS. The excess liquid was taken by a paper tissue and the weight was measured. Subsequently, the collagen was solubilized by adding 1 mL pepsin solution (0.5 mg mL^−1^ in 0.5 M acetic acid) at 4 °C for 24 h. To graph the standard curve, the same procedure was conducted to a blank (10 mg PGA‐fibrin unwoven meshwork in 0.1 mg mL^−1^ pepsin solution) and known collagen concentration of 5, 10, and 15 µg. The solutions were then neutralized by adding 100 µL neutralizing solution. Thereafter, a PEG solution was added for 24 h at 4 °C to concentrate collagen according to the manufacturer's protocol followed by samples centrifugation at 10 000 × *g* for 10 min to isolate the collagen pellets. To each tube, 1 mL Sircol dye reagent (sirius red) was added and mixed well for 30 min and then spun for 10 min at 10 000 × *g*. At this step, collagen will bind to sirius red dye and precipitate. To remove the unbounded dyes, the precipitate was washed with ice‐cold acid‐salt solution and centrifuged again to collect the pellets. Finally, the bonded dyes to collagen were dissolved in 250 µL of alkali reagent and transferred to 96‐well plate and measured at 555 nm with Tecan microplate reader (Switzerland). The collagen concentration was calculated based on standard curve and presented in micrograms per milligram of tissue weight.

##### Total Elastin Analysis

The Fastin Elastin Assay kit (Biocolor) was used to analyze the total elastin content of scaffolds and tissues according to the manufacturer protocol.^[^ ^70^ ^]^ Briefly, samples were weighed and digested in 0.25 M oxalic acid at 100 °C for 1 h. For animal specimens, tissues were disintegrated using an ultrasound homogenizer to improve the oxalic acid digestion. To draw the standard curve, a blank (PGA‐fibrin scaffold) and gradient of known concentration of soluble *α*‐elastin were processed similarly. All solutions were filtered by 0.21 µm syringe filter to minimize the effect of running particles in future steps. After elastin dissolution, it was precipitated at 4 °C for 15 min using precipitating reagent and centrifuged at 10 000 × *g* for 10 min to separate the pellets. Then the pellets were suspended in 1 mL dye reagent and mixed for 90 min followed by 10 min of centrifugation at 10 000 × *g* to separate the pellets. Finally, the pellets dissolved in 250 µL dye dissociation reagent and measured by microplate reader at 513 nm to calculate the elastin concentration. The elastin concentration was calculated based on the standard curve and presented in micrograms per milligram of tissue weight.

##### Fluorescence Immunostaining and Fibronectin Visualization

For fluorescence immunostaining, tissues were washed with PBS and then fixed in 4% paraformaldehyde in PBS for 30 min. Nonspecific adsorption of antibody was prevented by albumin preincubation with bovine serum albumin (2% w/v, 30 min). After washing the samples three times with PBS, tissues were incubated with primary antibodies according to Table S1, Supporting Information. Samples were then washed three times with Dulbecco's phosphate‐buffered saline (DPBS) and treated with the secondary antibody (goat anti‐rabbit Alexa Fluor 633 or goat anti‐mouse Alexa Fluor 633 all from Invitrogen) with dilution of 1:500 DPBS for 1 h at room temperature. Nonspecific antibody binding of primary and secondary antibodies was controlled by blank samples (negative control) or animal or human tissues specimens that present antigens (positive control) as exemplified for elastin in Figure S15, Supporting Information. Cell nuclei were stained by 4′,6‐diamidino‐2‐phenylindole (2 µg mL^−1^, 10 min). Samples were finally washed three times with PBS. For visualization of human plasma fibronectin, Alexa Fluor 488‐labeled human plasma fibronectin was added to the culture medium (5 µg mL^−1^) during the culture period as described before.^[^ ^71^ ^]^ Labeled fibronectin gets incorporated by cells into their fibrillar fibronectin ECM networks. The samples were imaged using a confocal microscope (Olympus FV1000, Japan).

##### Image Analysis

Pixel‐by‐pixel signal intensity analyses were performed using image histograms while keeping the imaging conditions identical for samples needing to be compared. Such analyses were performed to compare collagen type I, III elastin, and fibrin density in front and backsides of the tissues. The average mean intensities from selected parts of an image were measured using ImageJ to plot graphs as shown in different figures. It should be noticed that the autofluorescence of partially degraded PGA fibers seen in various images (after 3 weeks) can be excited and imaged at 633 nm with rather low laser intensity (Figure S16, Supporting Information). To overcome this issue, the parts of images were manually selected and analyzed in which there were no PGA fibers in the field of view as shown in Figure S17, Supporting Information. In this process, the fibronectin channel was used as a guide to choose areas with ECM on uneven surface of samples. For the representative images given in the figures, 20 images were stacked from the surface to 50 µm in depth to include and visualize maximum accessible surface. Cross‐sectional confocal images were taken by mounting the sample in vertical direction to the field of view.

##### RNA Extraction and Reverse Transcription

Total RNA was extracted using the Rneasy RNA isolation kit and column (Qiagen) following the manufacturer's instructions. Reverse transcription was performed for each sample in 50 µL reaction mixture containing 40 µL of RNA in RNAase‐free water (40 ng µL^−1^), 8 µL of iScript advanced reaction mix, and 2 µL of iScript Advanced reverse transcriptase all from Bio‐Rad. The conditions for the reverse transcription were as follows: 46 °C for 20 min, followed by 95 °C for 1 min.

##### Quantitative Real‐Time PCR

The resulting cDNA was amplified in duplicate by quantitative real‐time PCR in 10 µL reaction mixtures with 200 × 10^−9^ m of each specific primer and 1× Fast Syber Green qPCR Master Mix (Life Technologies). For the amplification reaction, QuantStudio 7 from Applied Biosystem was used. The amplification program was set as follows: 95 °C for 5 min, followed by 45 cycles at 95 °C for 10 s, 60 °C for 15 s, 72 °C for 20 s. The glyceraldehyde 3‐phosphate dehydrogenase and 18S gene expression was used for sample normalization and the Excel software was used for the comparative quantification analysis. The primers were designed using the free online software Primer3. The sequences were tested for their specificity by performing a BLAST alignment (basic local alignment search tool) on the human respective mRNA.

##### Statistics

Data were compared using one‐way ANOVA with post hoc Bonferroni test (OriginPro 9). Error bars if any are standard deviation unless noted in image caption.

## Conflict of Interest

The authors declare no conflict of interest.

## Supporting information

Supporting Information

Supplemental Movie 1

Supplemental Movie 2

Supplemental Movie 3

Supplemental Movie 4

Supplemental Movie 5

Supplemental Movie 6

Supplemental Movie 7

Supplemental Movie 8
