# Supplementary material for: A Pulsatile Flow System to Engineer Aneurysm and Atherosclerosis Mimetic Extracellular Matrix
Source: Adv Sci (Weinh). 2020 Apr 30;7(12):2000173. doi: 10.1002/advs.202000173 (PMC7312268; doi:10.1002/advs.202000173)
Supplement: Supplementary file 1 — Supporting Information [file ADVS-7-2000173-s001.pdf]

A Pulsatile Flow System to Engineer Aneurysm and Atherosclerosis Mimetic Extracellular Matrix

Vahid Hosseini+, Anna Mallone, Nima Mirkhani, Jerome Noir, Mehdi Salek, Francesco Silvio Pasqualini, Simone Schuerle, Ali Khademhosseini, Simon P. Hoerstrup, Viola Vogel**

Corresponding Authors: Dr. V. Hosseini, Prof. V. Vogel
Laboratory of Applied Mechanobiology, Institute of Translational Medicine, Department of Health Sciences and Technology, ETH, 8093 Zurich, Switzerland

Dr. A. Mallone, Dr. F. S. Pasqualini, Prof. S. P. Hoerstrup
Institute for Regenerative Medicine (IREM), University of Zurich and Wyss Translational Center Zurich, 8952 Zurich, Switzerland

Nima Mirkhani, Prof. Simone Schuerle
Responsive Biomedical Systems Lab, Institute of Translational Medicine, Department of Health Sciences and Technology, ETH, 8093 Zurich, Switzerland

Dr. M. Salek
Department of Mechanical Engineering, Massachusetts Institute of Technology, Boston, MA 02139, USA

Dr. J. Noir
Institute of Geophysics, Department of Earth Sciences, ETH Zurich, 8092 Zurich, Switzerland

Dr. F. S. Pasqualini
Synthetic Physiology Laboratory, Department of Civil Engineering and Architecture, University of Pavia, 27100 Pavia, Italy

Prof. Ali Khademhosseini
Department of Bioengineering, University of California-Los Angeles, Los Angeles, CA 90095, USA

+ Current address: Department of Bioengineering, University of California-Los Angeles, Los Angeles, CA 90095, USA

***Corresponding Authors:** Vahid Hosseini, svhosseyni@g.ucla.edu and Viola Vogel, viola.vogel@hest.ethz.ch

Supplemental Figures and Tables

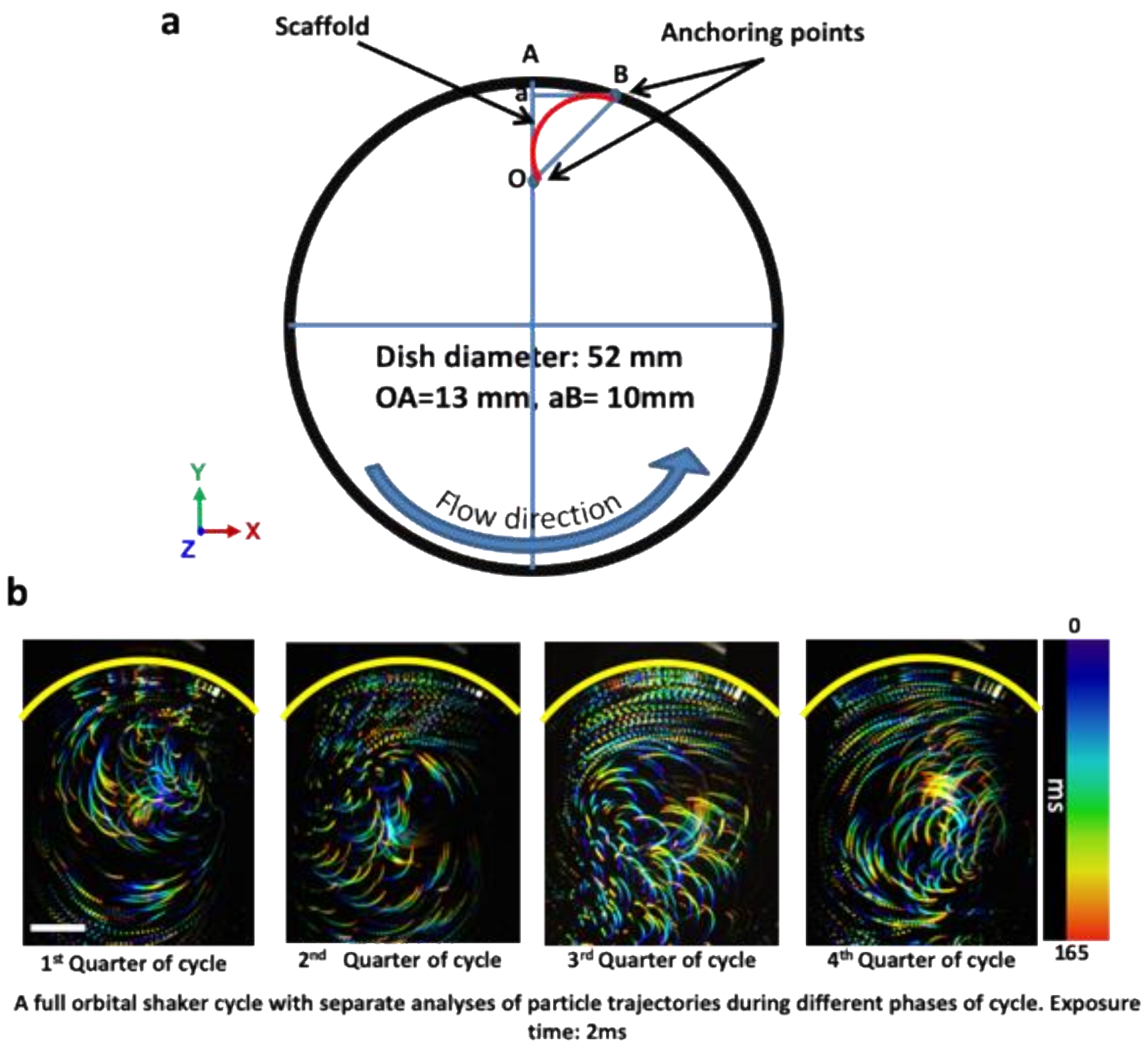

Figure S1: (a) Configuration of device with detailed plan for mounting a scaffold to minimize the geometrical variation in the experimental setups. (b) XY Trajectories of sloshing flow in the dish without obstacle. The tangential flow near the dish wall is a laminar pulsatile flow. One cycle was visualized in 4-quarter cycle for ease of visibility. Dish diameter was 5 cm and frequency of shaker was 1.5 Hz. (c) Plot of the flow velocity profile during 3 cycles was experimentally quantified by measuring the particle trajectories (fluorescent microspheres) near the front and backsides of the tissue and its comparison to CFD analysis of flow velocity at the wall. Scale bar 5 mm. Supplemented to Figure 1.

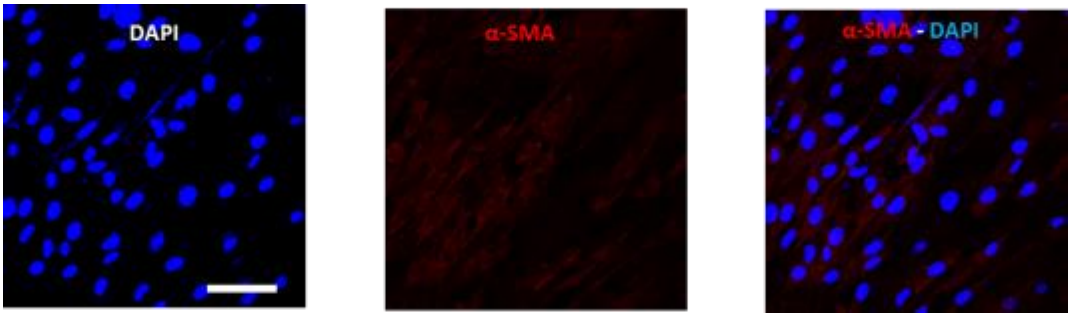

Figure S2: Human primary vascular smooth muscle cells during the expansion on plastic culture dish (static condition) do not express αSMA marker and are transitioning to a fibroblastic phenotype. Nuclei stained with DAPI (blue) and αSMA stained with primary antibody against αSMA then 633-Alexaflour secondary antibody (red). Scale bar 50 μm. Supplemented to Figure 3.

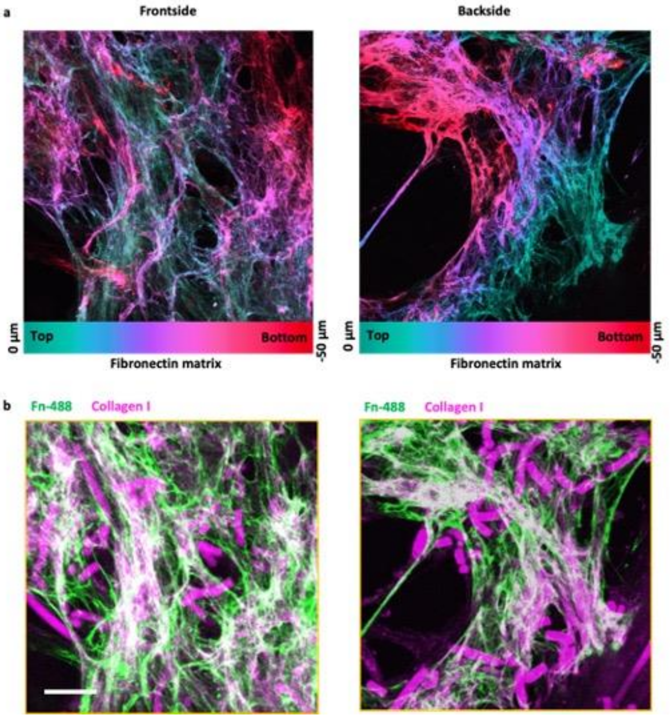

Figure S3: Two representative tissue image stacks from figure 6c. (a) Top-down spatial color-coded map of assembled fibronectin, going from the surface of the tissue 50 μm deep into the core. (b) The same image with collagen I immunostaining shows the unevenness of tissue surface both in different flow conditions and in presence of doxycycline. The tissues immunostained after three weeks in culture for collagen I (magenta). Fibronectin (green) was visualized not by immunostaining, but by supplementing labeled AlexaFlour 488 human plasma fibronectin (green) to the cell culture medium. Scale bar 50 μm.

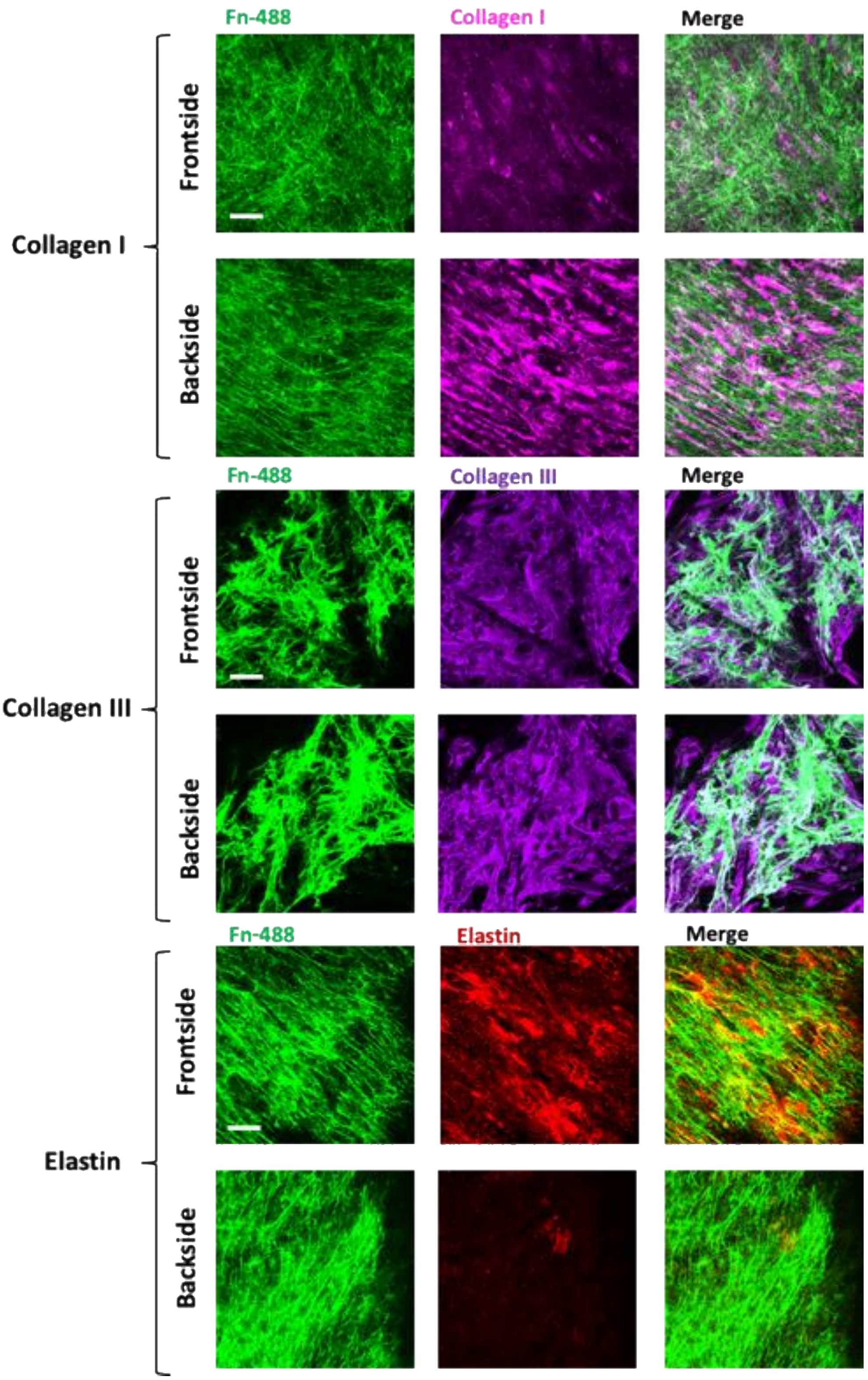

Figure S4: Differential deposition of collagen I, III and elastin in the outer surface layers of the engineered tissues that faced toward different flow regimes. Representative z-stack maximum projection intensity images of the front and backsides of the tissues, which were shown in merge mode in Figure 4. The tissues were immunostained after three weeks in culture for collagen I (magenta), collagen III (violet), and elastin (red). Fibronectin was visualized not by immunostaining, but by supplementing labeled AlexaFlour 488 fibronectin (green) to the cell culture medium (supplemented to Figure 4). Scale bar 50 µm.

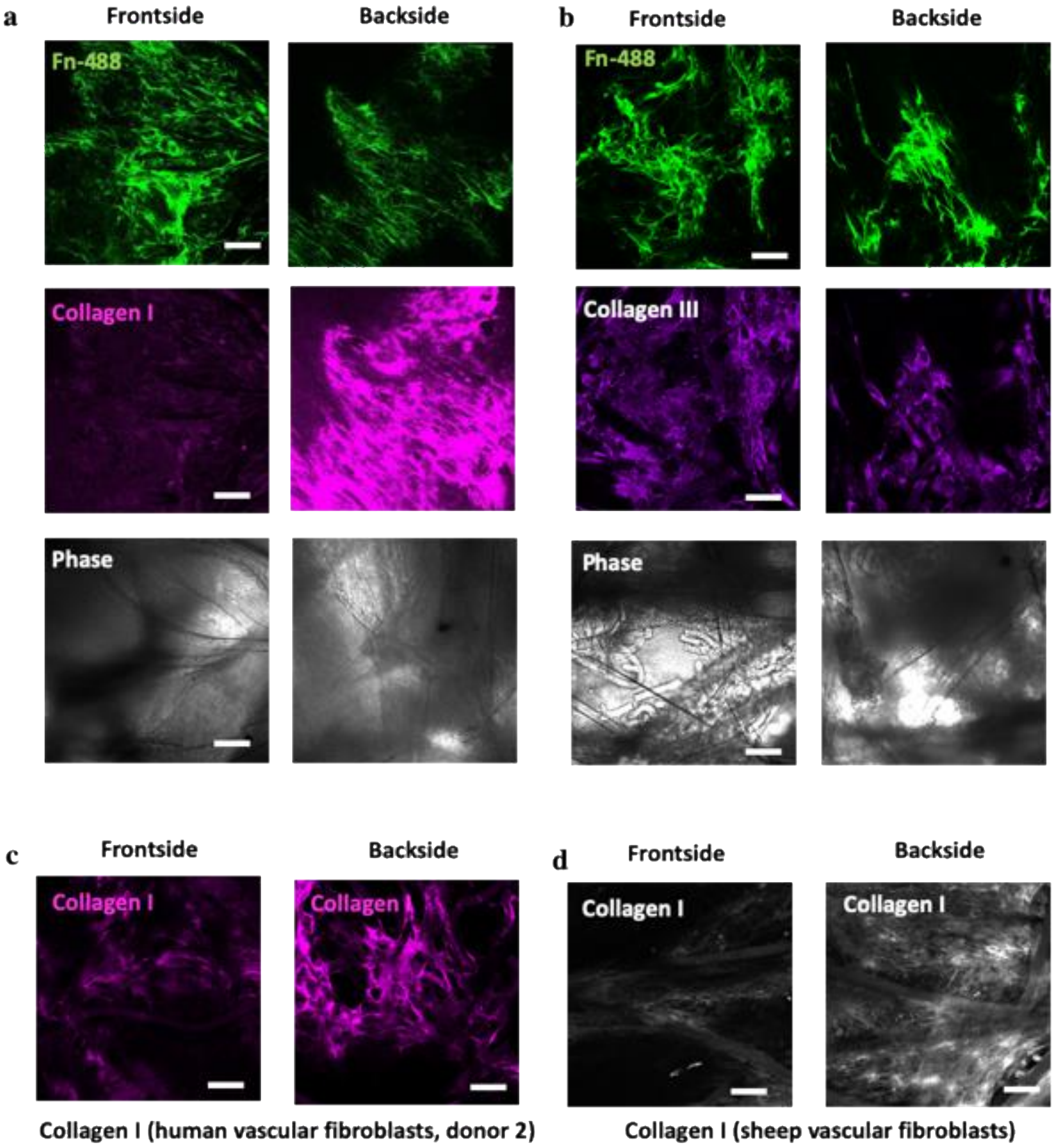

Figure S5: Collagen I, III matrix synthesis under the two different flow conditions was visualized by immunostaining. (a) Representative single stack confocal images on front or back of tissues which were immunostained and analyzed either for collagen I (2th Ab AlexaFlour 633, magenta) and collagen III (violet) under identical microscopy setup. Fibronectin was visualized by supplementing the culture medium with labeled AlexaFlour 488 fibronectin (green) throughout the experiment. As the tissues had uneven surfaces, fibronectin was used as guide to identify the planes used for data analysis. (b) Cross sectional images of same tissue captured by vertical placement of tissue toward the objective lens (10x). Immunostaining of (c) collagen I of human SMCs (donor 2), and (d) collagen I of sheep SMCs. Scale bars (a, c, d, e) 50 µm and (b) 100 µm. Supplemented to Figure 4.

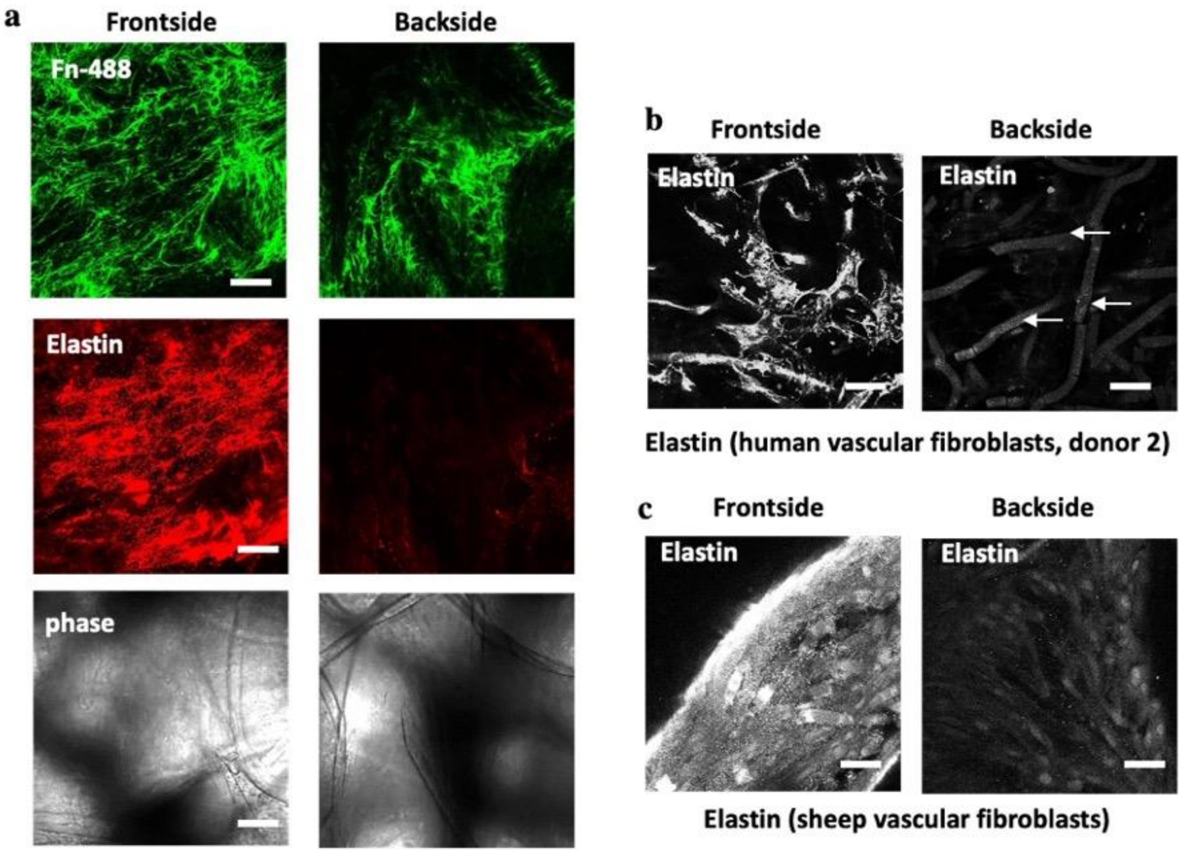

Figure S6: Elastin matrix synthesis in the front and backside of the flap was visualized by immunostaining. (a) Representative confocal images of the front or backsides of the engineered tissues in different flow conditions which were immunostained and analyzed for elastin (2th Ab AlexaFlour 633, red), both under identical microscopy setups. Fibronectin was visualized by supplementing the culture medium with AlexaFlour-488 labeled human plasma fibronectin (green) throughout the experiment. As the tissues had uneven surfaces, fibronectin was used as guide to identify the planes used for data analysis. Immunostaining of (b) elastin of human SMCs (donor 2), and (c) elastin of sheep SMCs. Arrows show the PGA scaffold fibers. Scale bars 50 µm.

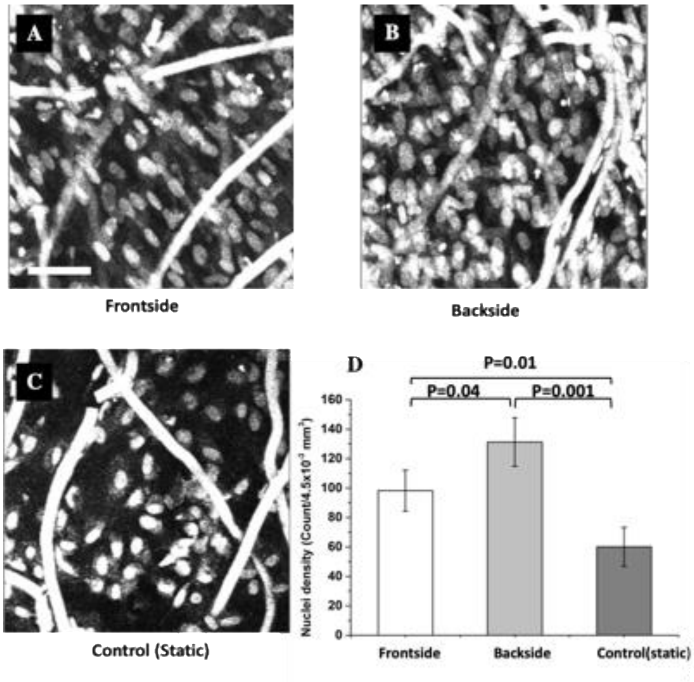

Figure S7: Cell nuclei density analysis in (a) front or (b) backside of the tissues exposed to different flow conditions or (c) static condition and (d) its comparative analysis. Cell nuclei was visualized by DAPI staining. Notice autofluorescence from the thick straight PGA scaffold fibers. Scale bar 50 µm.

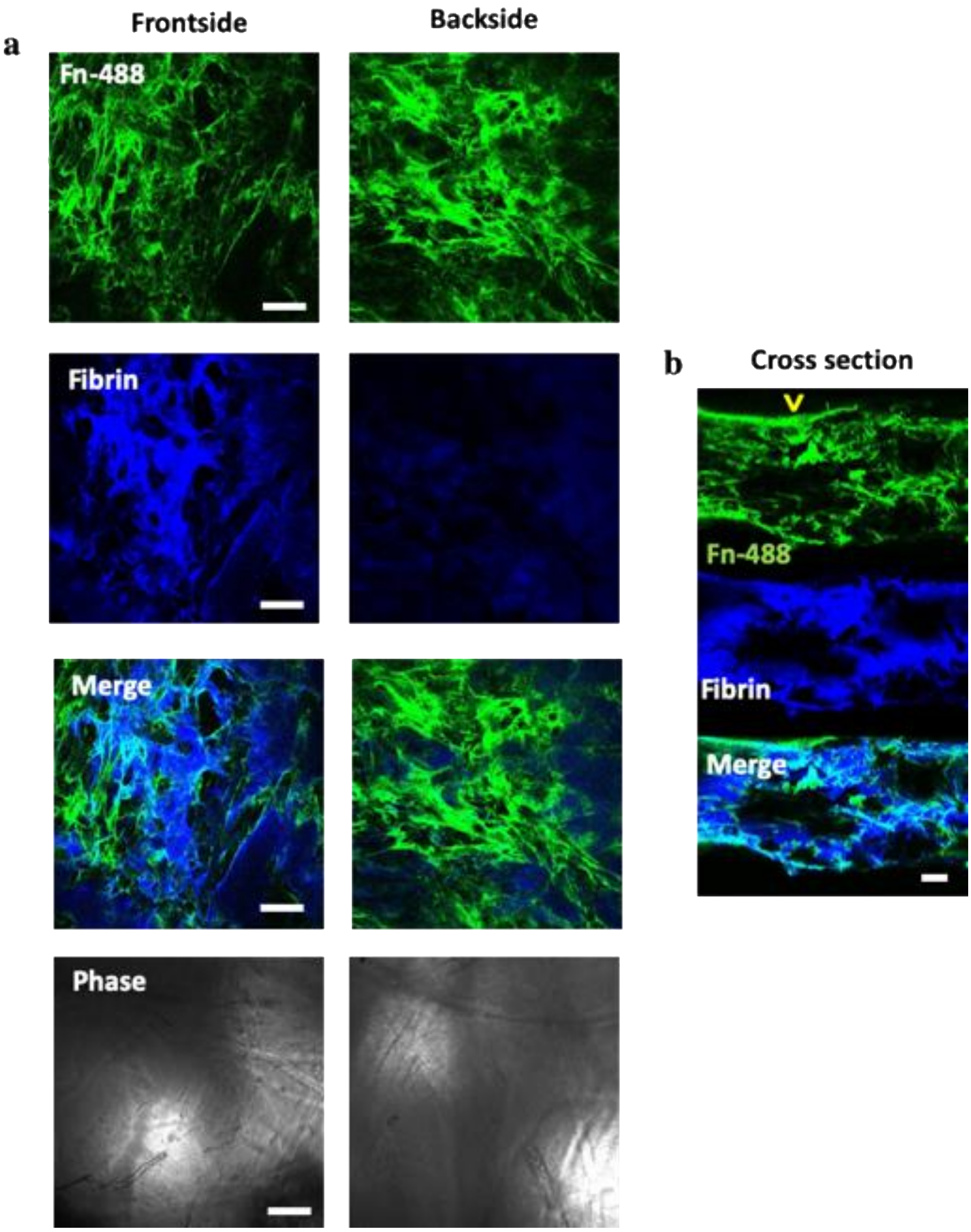

Figure S8: Fibrin degradation under the influence of the two flow conditions was visualized by immunostaining. (a) Representative confocal images of front and backside of tissue which were immunostained and analyzed for fibrin (2th Ab AlexaFlour 633, blue). Fibronectin was visualized by supplementing the culture medium with labeled AlexaFlour 488 fibronectin (green) throughout the experiment. As the tissues had uneven surfaces, fibronectin was used as guide to identify the planes used for data analysis. (b) Cross sectional images of the same tissue captured by vertical placement of tissue toward the objective lens (10x). Scale bars (a) 50 µm and (b) 100 µm. Supplemented to figure 5.

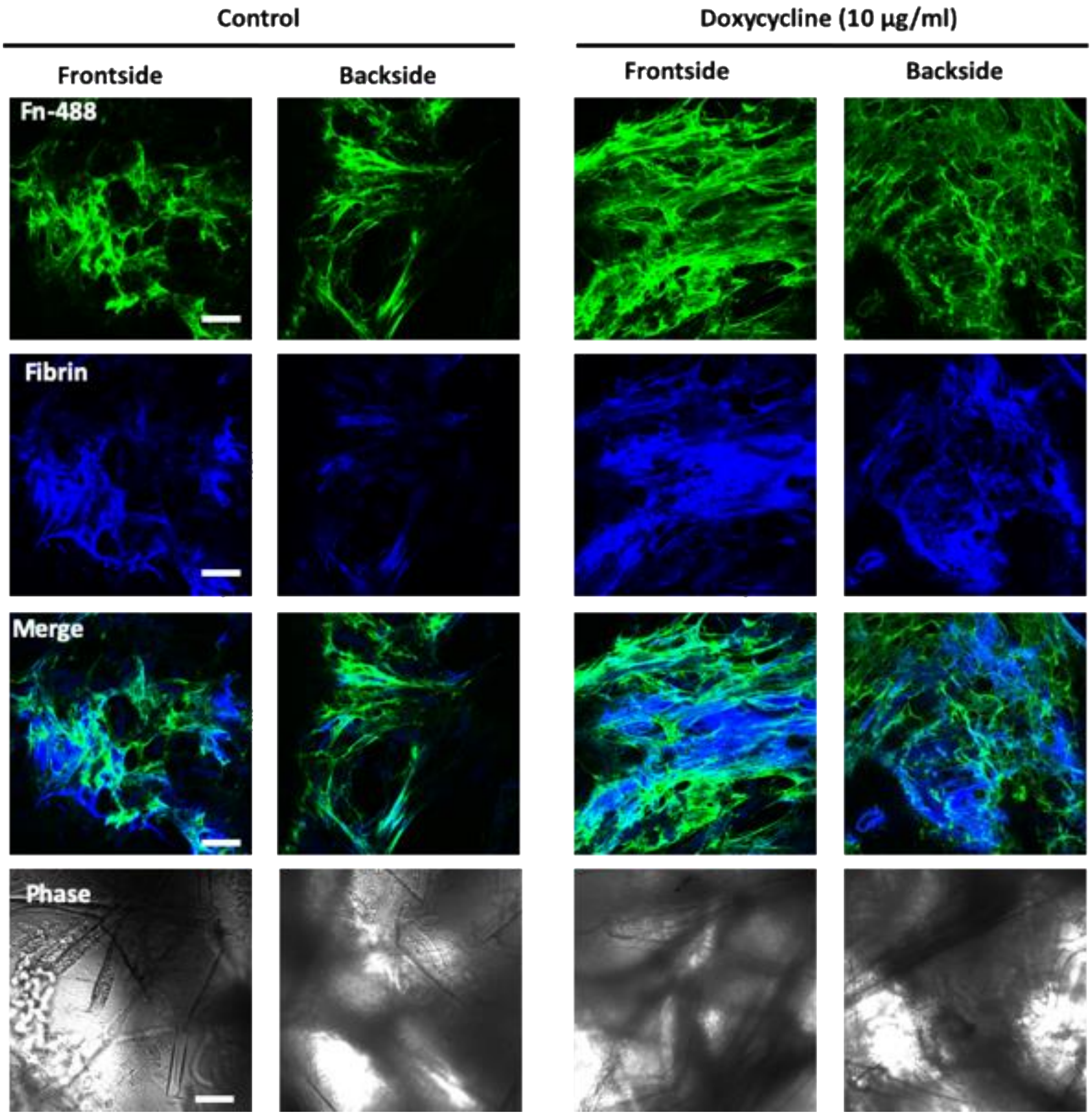

Figure S9: Fibrin degradation was slowed down by doxycycline. Comparative immunostaining with identical confocal microscopy setups showed slower fibrin degradation compared to non-treated samples particularly on backside of tissues after 3 weeks of SMCs culture with last 11 days in presence of 10µg/ml doxycycline. Fibronectin was visualized by supplementing the culture medium with labeled AlexaFlour 488 fibronectin (green) throughout the experiment. As the tissues had uneven surfaces, fibronectin was used as guide to identify the planes used for data analysis. Scale bars 50 µm.

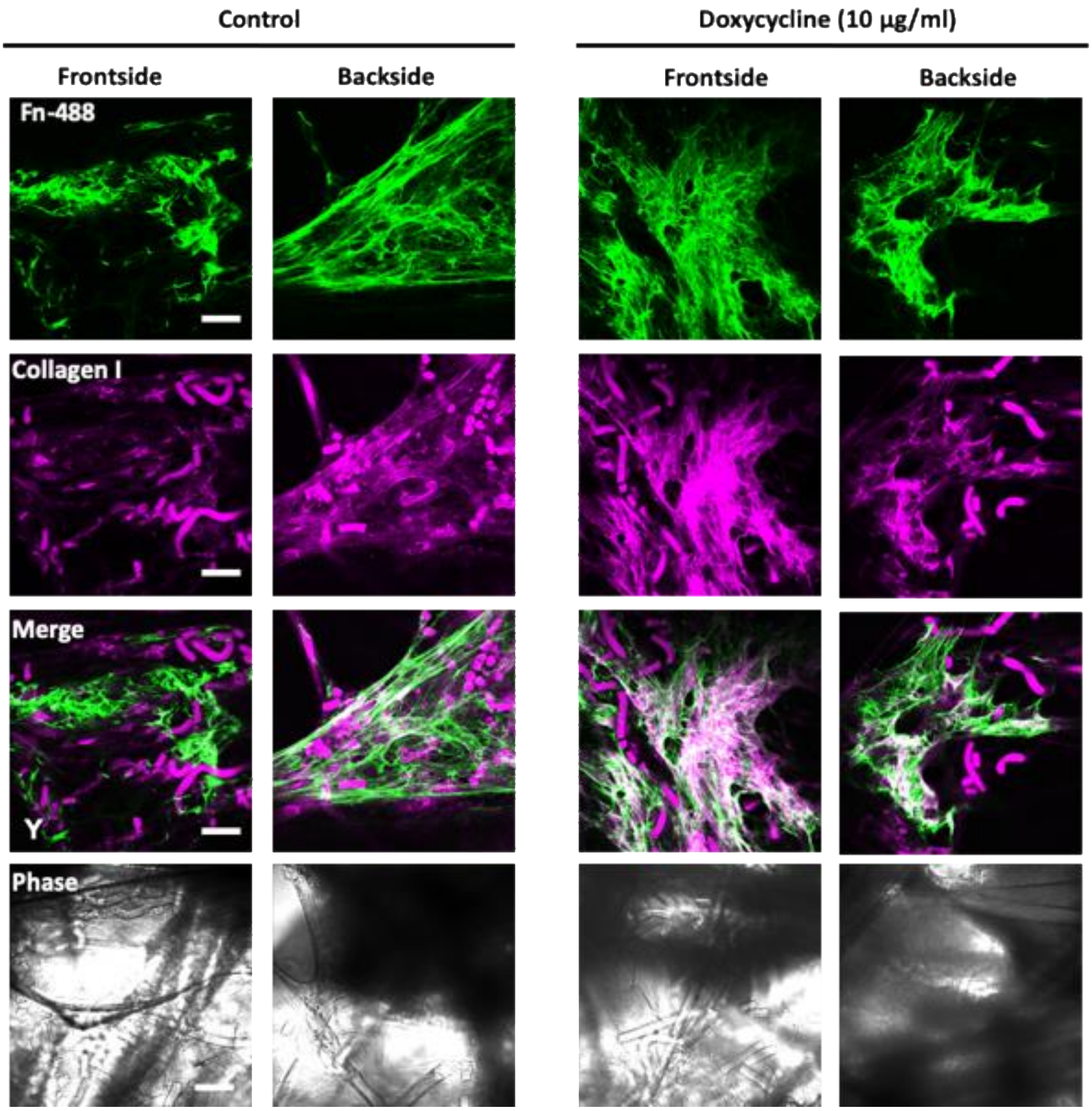

Figure S10: Collagen I assembly was enhanced by doxycycline on the frontside. Comparative immunostaining with identical confocal microscopy setups showed higher collagen I (magenta) was assembled on Doxycycline treated tissues compared to non-treated tissues particularly on the frontside of tissues after 11 days of SMCs culture in presence of 10µg/ml doxycycline. Fibronectin was visualized by supplementing the culture medium with labeled AlexaFlour 488 fibronectin (green) throughout the experiment. As the tissues had uneven surfaces, fibronectin was used as guide to identify the planes used for data analysis. Notice autofluorescence from the thick straight PGA scaffold fibers. Scale bars 50 µm.

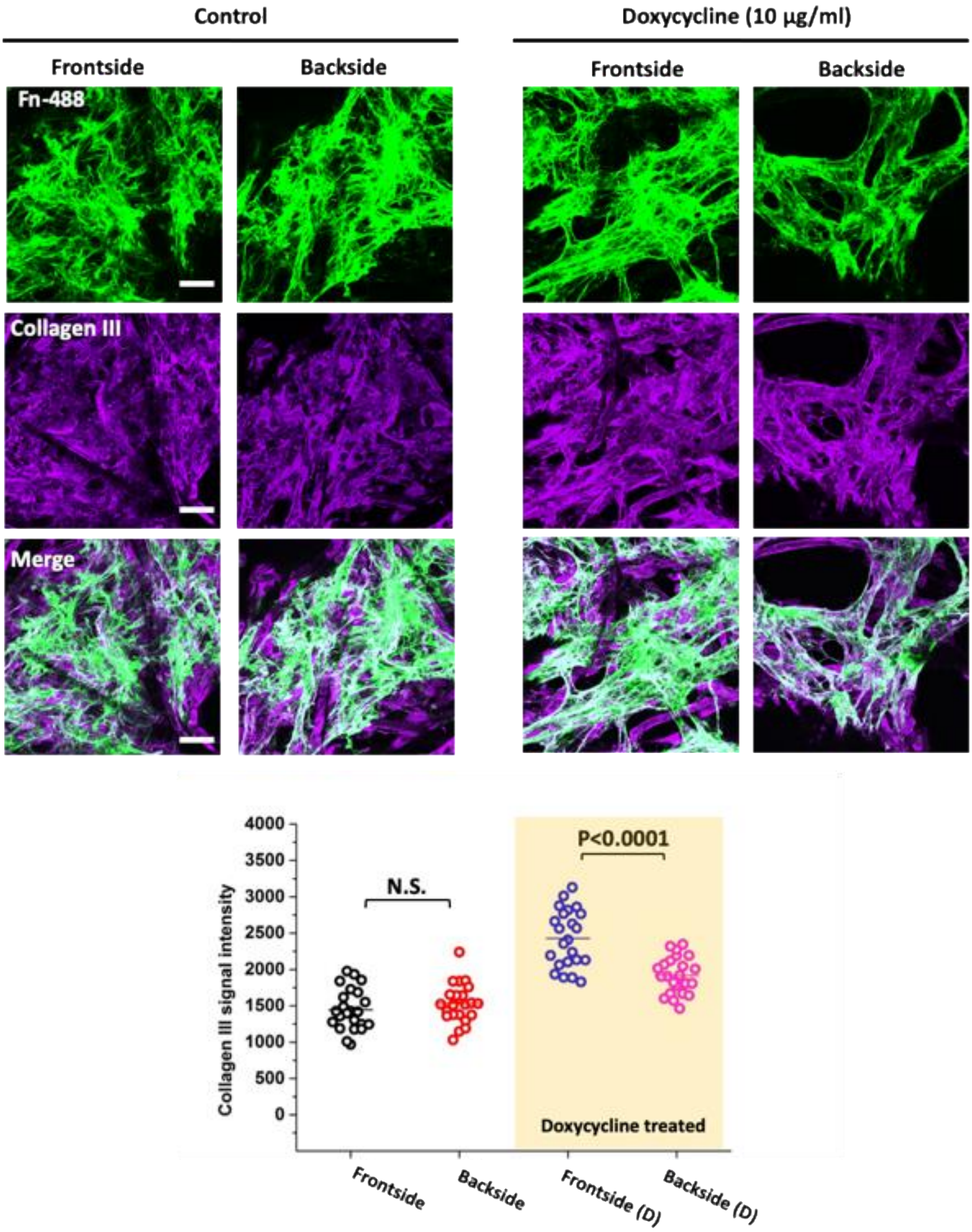

Figure S11: Collagen III assembly was enhanced by doxycycline, particularly on the frontside. Comparative immunostaining showed higher collagen III (red) was assembled in Doxycycline treated tissues compared to non-treated tissues. Significantly higher collagen III was assembled on the frontside after 11 days of treatment with 10µg/ml Doxycycline. Fibronectin was visualized by supplementing the culture medium with labeled AlexaFlour 488 fibronectin (green) throughout the experiment. As the tissues had uneven surfaces, fibronectin was used as guide to identify the planes used for data analysis. Scale bars 50 µm.

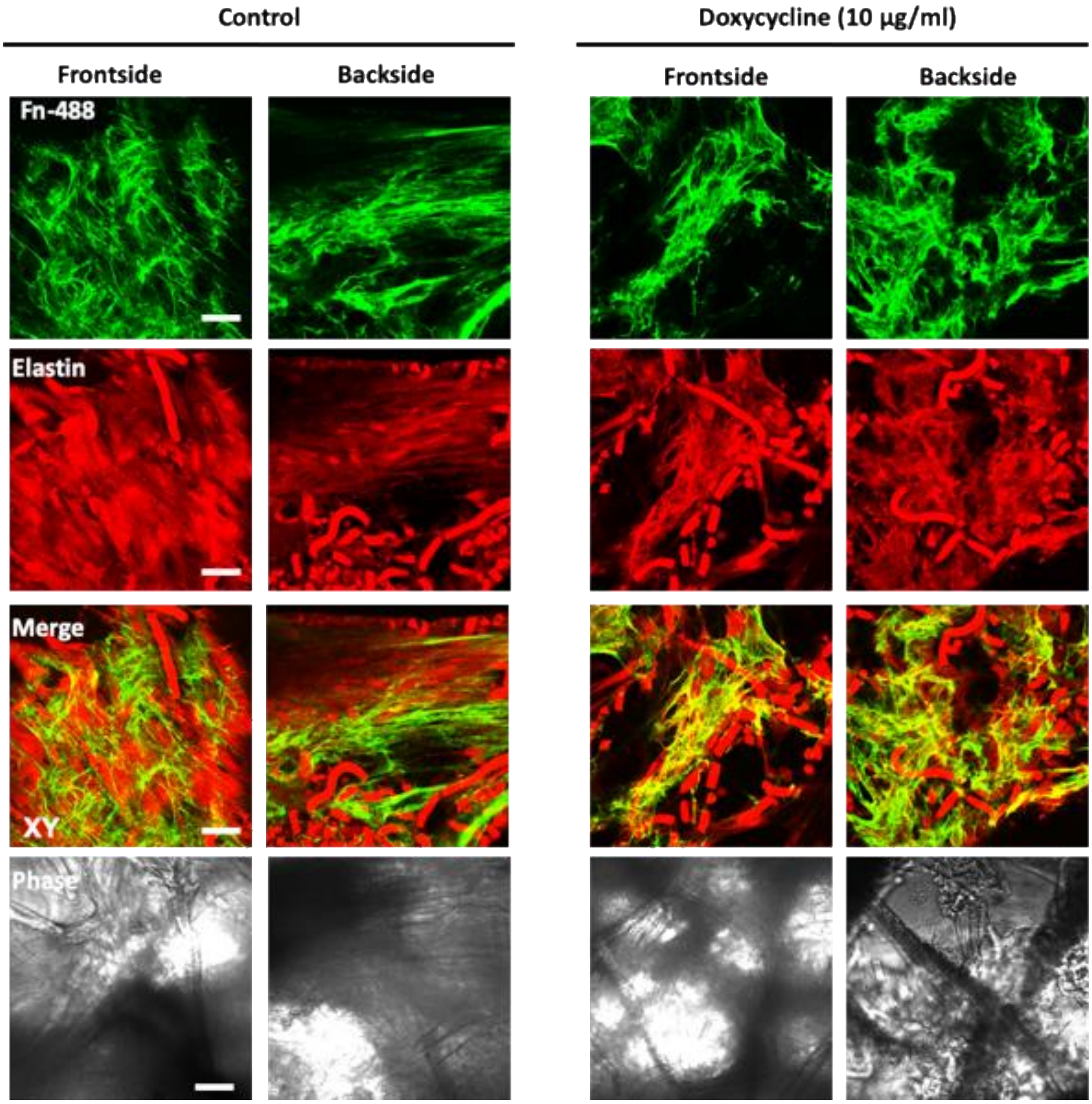

Figure S12: Elastin assembly was enhanced on the backside of tissues after Doxycycline treatment. Comparative immunostaining showed that a higher elastin content (red) were assembled in Doxycycline treated tissues compared to non-treated samples particularly on the backside of tissue after 11 days of treatment with 10µg/ml doxycycline. Fibronectin was visualized by supplementing the culture medium with labeled AlexaFlour 488 fibronectin (green) throughout the experiment. As the tissues had uneven surfaces, fibronectin was used as guide to identify the planes used for data analysis. Notice autofluorescence from the thick straight PGA scaffold fibers. The signal from the fibers were eliminate from analysis as described in method section and Figure S17. Scale bars 50 µm.

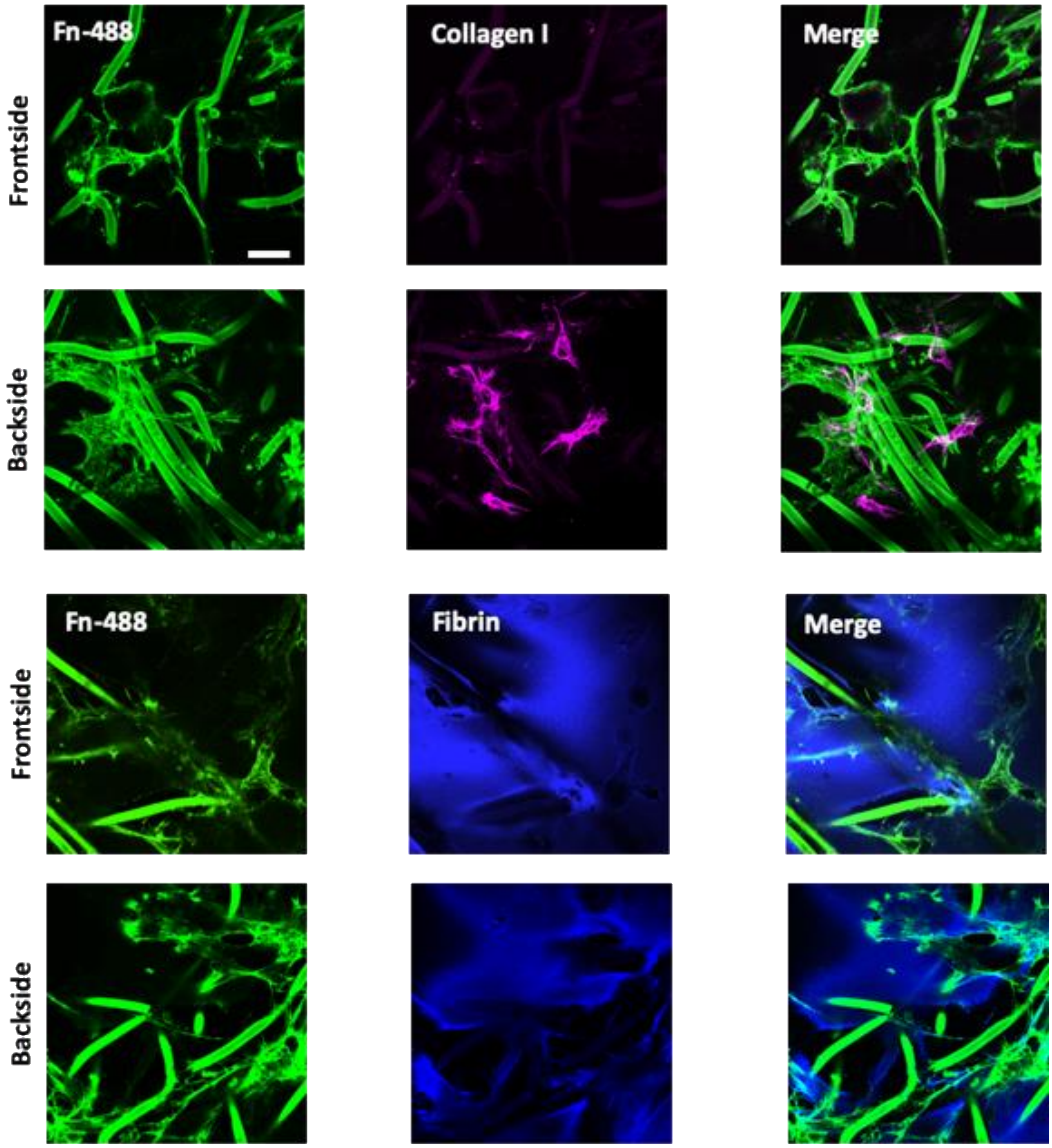

Figure S13: The effect of high dose doxycycline on cell matrix synthesis and fibrin degradation. Addition of doxycycline (40 µg/ml) from the starting day of culture, inhibits the cells to degrade fibrin matrix and new matrix assembly. The confocal images captured after 21 days cell culture under different flow regimes is showing almost intact fibrin gel (blue), limited fibronectin (green) and collagen I (magenta) matrix assembly. Interestingly, the backside of tissues showed higher collagen I deposition compared to the frontside. Notice autofluorescence from the thick straight PGA scaffold fibers. Scale bar 50 µm.

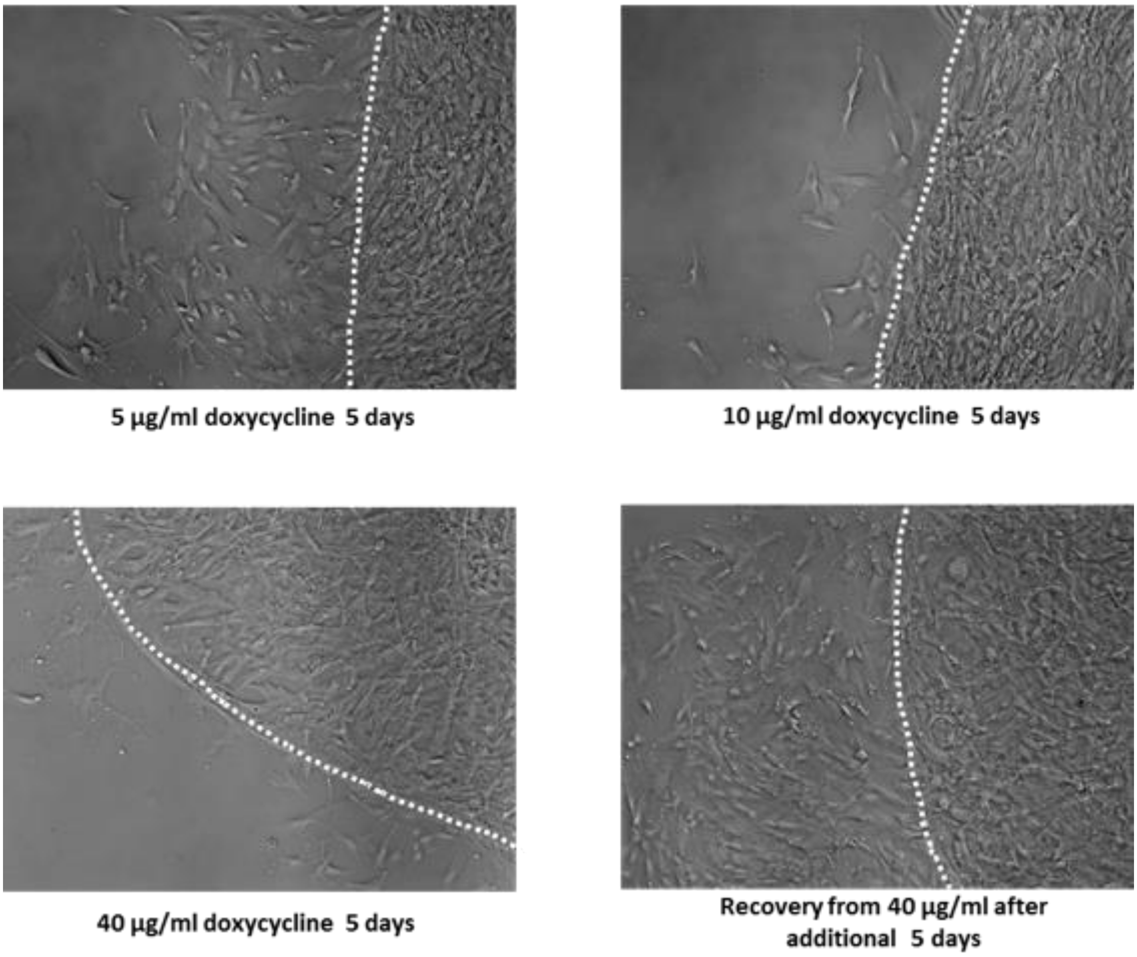

Figure S14: Dose dependent inhibitory effect of Doxycycline on migration of SMCs from fibrin gel. Phase contrast images show encapsulated cells in fibrin hydrogel migrated less out of the gel when treated with different doses of Doxycycline (Dash-lines show the borders of fibrin gels). However, a low dose of doxycycline (5 µg/ml) is less effective compared to high doses and removal of the drug could recover cell migration and fibrin gel degradation.

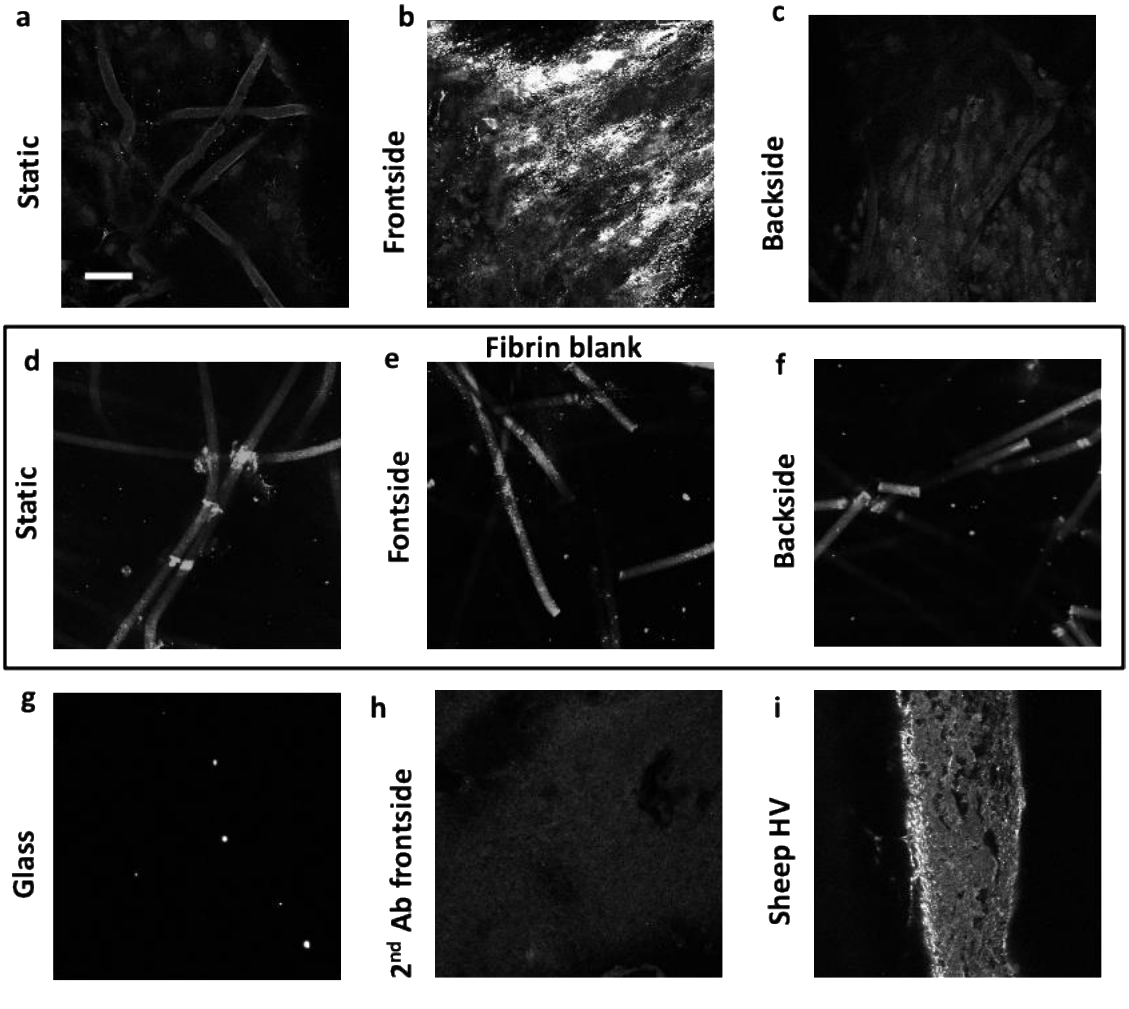

Figure S15: Immunostaining and validation of elastin on different samples with positive and negative controls. Images were taken with the same identical parameter for comparative analysis on (a) static condition (b) frontside of cultured tissue and (c) backside. (d-f) PGA-Fibrin tissues without cells cultured for 3 weeks were immunostained as negative control, showed up only PGA fibers. (g) BSA coated glass immunostained for elastin as blank control. (h) Secondary antibody Alexaflour 633 was used for staining of frontside of tissue to control unspecific antibody adsorption and (i) cross section of sheep heart valve leaflet as positive control (left side of images show ventricular side). Notice autofluorescence from the thick straight PGA scaffold fibers. Scale bar 50 µm.

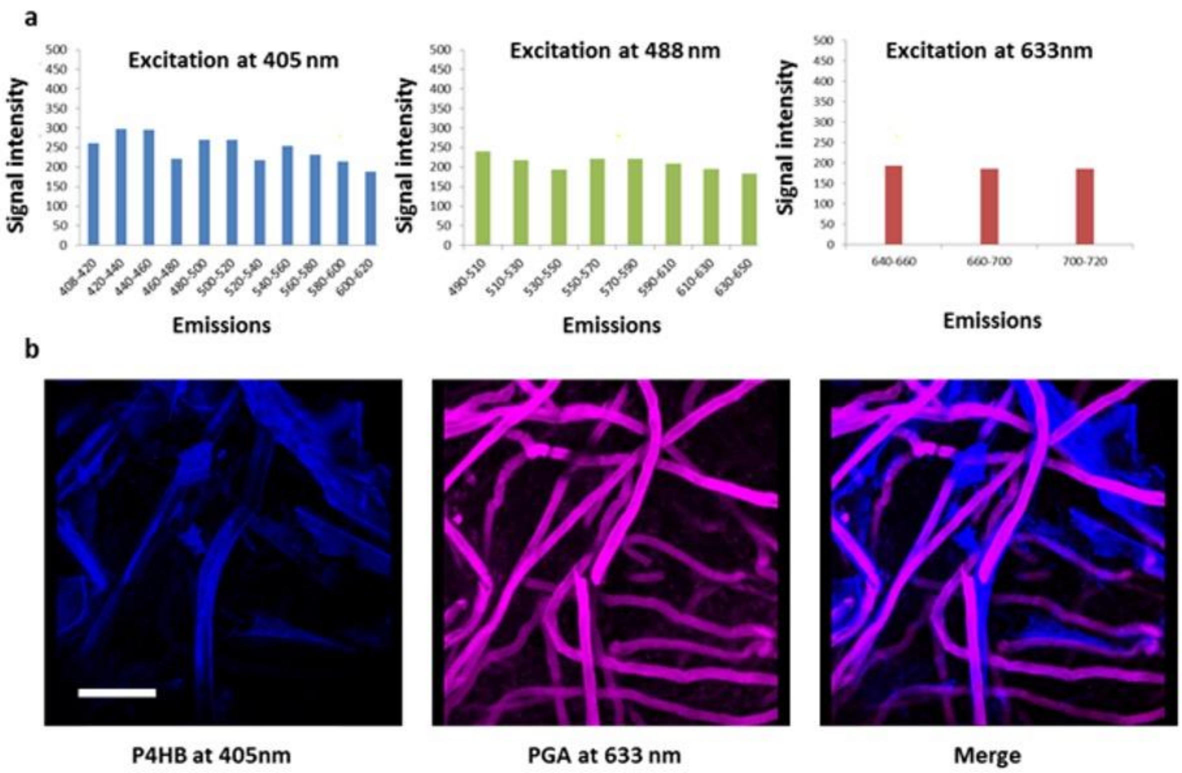

Figure S16: PGA autofluorescence. (a) Excitation-emission graph of PGA at different wavelengths. (b) 3D maximum projection intensity of P4HB (used for coating of PGA scaffold fibers) autofluorescence captured at 405 nm and PGA at 633 nm after 3 weeks of conditioning in culture medium (37 °C) showed that the laser excitation at 633 nm can visualize the moderately degraded PGA fibers. Scale bar 50 μm.

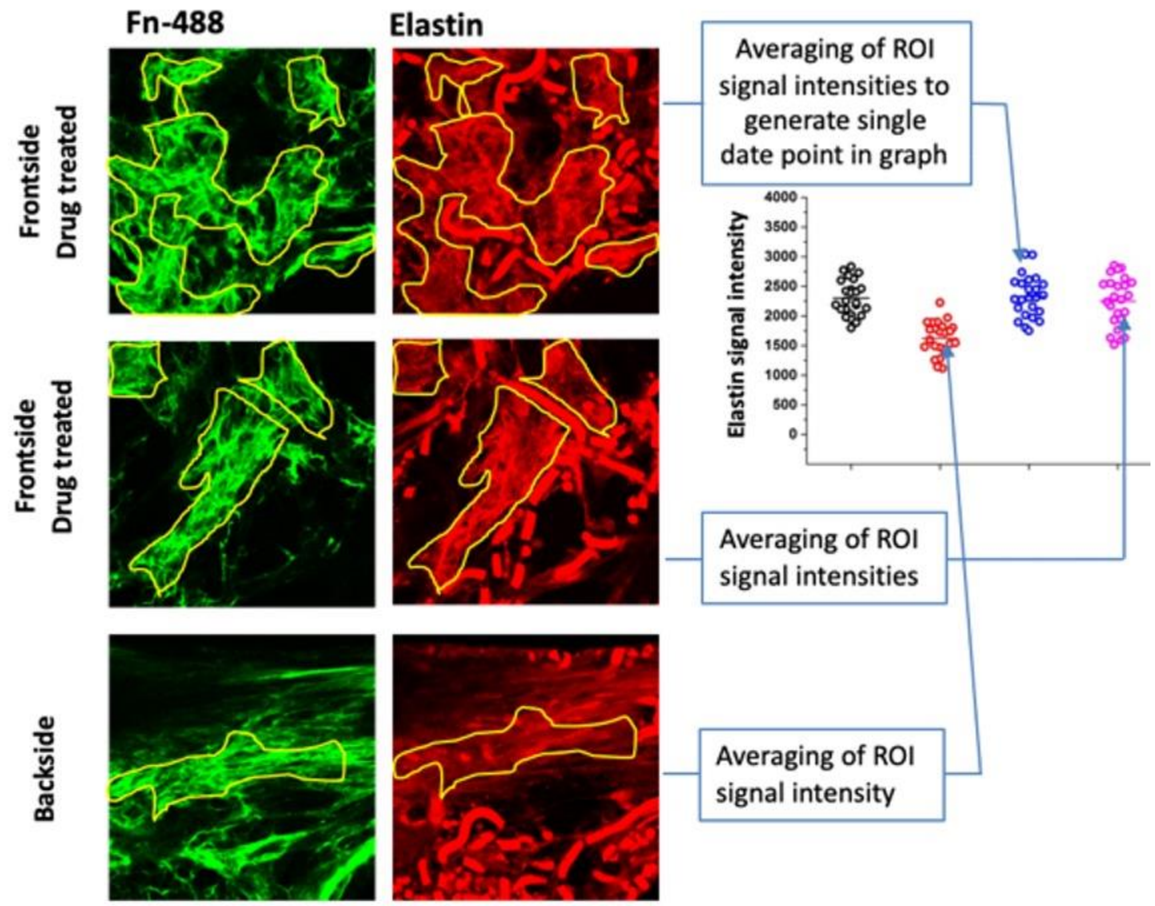

Figure S17: Method to correct for the autofluorescence of the scaffold PGA fibers for comparative signal analysis of confocal images. Samples were immunostained for the protein of interest (in this case elastin and 2th Ab AlexaFlour 633, red). Fibronectin was visualized by supplementing the culture medium with labeled AlexaFlour 488 fibronectin (green) throughout the experiment. As the tissues had uneven surfaces, fibronectin was employed as guide to identify the planes (ROI, encircled by yellow) and used for data analysis. The average signal intensity of every pixel in ROI is the source of every data point in the graph. For comparison, the data points were statistically compared using one-way ANOVA with post hoc Bonferroni test (Origin-Pro 9). PGA autofluorescence were excluded from image analysis manually by choosing ROIs. Images scale as exhibited in image S12.

Table 1: List of primary antibodies used in this study

Antibody against	Vendor, Catalog #	Dilution	Incubation time
Alpha SMA	Abcam, ab7817	1/100 in PBS	1 hour at room temp
Collagen I	Abcam, ab 90395	1/50 in DMEM	1 hour at 37 C
Collagen III	Abcam, ab7778	1/100 in PBS	1 hour at room temp
Elastin	Abcam, ab23747	1/100 in PBS	1 hour at room temp
Fibrinogen	Sigma-Aldrich, F9902	1/100 in PBS	Overnight at 4 C
MMP2	Abcam, ab37150	1/100 in PBS	1 hour at room temp
MMP12	Abcam, ab137444	1/100 in PBS	1 hour at room temp

Table 2: List of Primers used in this study

**Gene**	**Forward Sequence (5'->3')**	**Reverse Sequence (5'->3')**
GAPDH	**GCG GGG CTC TCC AGA ACA TCA**	**GAC GCC TGC TTC ACC ACC TTC TT**
18S	**CCC GGG GAG GTA GTG ACG AAA AAT**	**GCC CGC TCC CAA GAT CCA ACT AC**
COL1A1	**GAA GGG CCA CGA CAA AGC AGA AAC**	**CCC CAC CCC ACC CAT CAC ATA G**
COL2A1	**TGGACGATCAGGCGAAACC**	**GCTGCGGATGCTCTCAATCT**
COL3A1	**GGAGCTGGCTACTTCTCGC**	**GGGAACATCCTCCTTCAACAG**
ELN	**GCA GGA GTT AAG CCC AAG G**	**TGT AGG GCA GTC CAT AGC CA**
MMP2	**GGA CAA AGG ATA CAA CAG GGA CCA AT**	**CCC AGG GAG TGG CCA ATT TCA T**
MMP9	**CGCTGGGCTTAGACTATTCCTCAGT**	**GATGCCATTCACGTCGTCCTTATG**
MMP12	**CATGAACCGTGAGGATGTTGA**	**GCATGGGCTAGGATTCCACC**
MMP13	**CGT ATT GTT CGC GTC ATG CCA G**	**TCT TCC CCT ACC CCG CAC TTC T**
MMP14	**CCCAGCCCACCCATTGAAGTCT**	**CCCGACATCCCTCTCCTCTGGC**
TIMP-1	**TGGAAAACTGCAGGATGGACTCTTG**	**CAGGGGATGGATAAACAGGGAAACA**
TIMP-2	**AAGCGGTCAGTGAGAAGGAAG**	**GGGGCCGTGTAGATAAACTCTAT**
TIMP-3	**CATGTGCAGTACATCCATACGG**	**CATCATAGACGCGACCTGTCA**
a-SMA	**AAAAGACAGCTACGTGGGTGA**	**GCCATGTTCTATCGGGTACTTC**

Movie captions:

Movie S1: Trajectories of moving fluorescent microspheres with the flow in a cell culture dish without obstacle. The tangential flow in the vicinity of the dish wall is laminar. The dish diameter was 5 cm and the frequency of the shaker was 1.5 Hz. Exposure time 25 ms, captured at 38 frames/s.

Movie S2: Trajectories of moving fluorescent microspheres with the flow in a cell culture dish with a mounted scaffold. The tangential flow in the vicinity of the dish wall is laminar. The dish diameter was 5 cm and the frequency of the shaker was 1.5 Hz. Exposure time 25 ms, captured at 38 frames/s.

Movie S3: CFD generated movie of flow velocity and color-coded contour at air/liquid interface. The dish diameter was 5 cm and the frequency of the shaker was 1.5 Hz in the model.

Movie S4: Slow motion movie of the petri dish with a drop of ink to visualize the fluid motion around the cell culture dish. Dish diameter was 5 cm and the frequency of the shaker was 1.5 Hz. Video was captured at 120 frame/S and playback at 24 frame/S.

Movie S5: CFD generated movie of flow shear stress along the X direction on the frontside. The dish diameter was 5 cm and the frequency of the shaker was 1.5 Hz in the model.

Movie S6: CFD generated movie of flow shear stress along the X direction on the backside. The dish diameter was 5 cm and the frequency of the shaker was 1.5 Hz in the model.

Movie S7: CFD generated movie of flow shear stress along the Y direction on the frontside. The dish diameter was 5 cm and the frequency of the shaker was 1.5 Hz in the model.

Movie S8: CFD generated movie of flow shear stress along the Y direction on the backside. The dish diameter was 5 cm and the frequency of the shaker was 1.5 Hz in the model.
